# Cryo-EM structures of lipopolysaccharide transporter LptB_2_FGC in lipopolysaccharide or AMP-PNP-bound states reveal its transport mechanism

**DOI:** 10.1038/s41467-019-11977-1

**Published:** 2019-09-13

**Authors:** Xiaodi Tang, Shenghai Chang, Qinghua Luo, Zhengyu Zhang, Wen Qiao, Caihuang Xu, Changbin Zhang, Yang Niu, Wenxian Yang, Ting Wang, Zhibo Zhang, Xiaofeng Zhu, Xiawei Wei, Changjiang Dong, Xing Zhang, Haohao Dong

**Affiliations:** 1 State Key Laboratory of Biotherapy and Cancer Center, National Clinical Research Center for Geriatrics, West China Hospital, Sichuan University and Collaborative Innovation Center of Biotherapy, 610041 Chengdu, China; 2 Department of Pathology of Sir Run Run Shaw Hospital, and Department of Biophysics, Zhejiang University School of Medicine, 310058 Hangzhou, Zhejiang China; 3 Center of Cryo Electron Microscopy, Zhejiang University, 310058 Hangzhou, Zhejiang China; 4 Key Laboratory of Combinatorial Biosynthesis and Drug Discovery, School of Pharmaceutical Sciences, Wuhan University, 430071 Wuhan, China; 5 College of Life Science, Sichuan University, 610041 Chengdu, China; 6 Biomedical Research Centre, Norwich Medical School, University of East Anglia, Norwich Research Park, Norwich, NR4 7TJ UK

**Keywords:** Membrane proteins, Biophysics, Microbiology, Molecular biology, Cryoelectron microscopy

## Abstract

Lipopolysaccharides (LPS) of Gram-negative bacteria are critical for the defence against cytotoxic substances and must be transported from the inner membrane (IM) to the outer membrane (OM) through a bridge formed by seven membrane proteins (LptBFGCADE). The IM component LptB_2_FG powers the process through a yet unclarified mechanism. Here we report three high-resolution cryo-EM structures of LptB_2_FG alone and complexed with LptC (LptB_2_FGC), trapped in either the LPS- or AMP-PNP-bound state. The structures reveal conformational changes between these states and substrate binding with or without LptC. We identify two functional transmembrane arginine-containing loops interacting with the bound AMP-PNP and elucidate allosteric communications between the domains. AMP-PNP binding induces an inward rotation and shift of the transmembrane helices of LptFG and LptC to tighten the cavity, with the closure of two lateral gates, to eventually expel LPS into the bridge. Functional assays reveal the functionality of the LptF and LptG periplasmic domains. Our findings shed light on the LPS transport mechanism.

## Abstract (web summary)

Seven lipopolysaccharide (LPS) transport proteins (LptBFGCADE) mediate the transport of LPS from the inner to the outer membrane of Gram-negative bacteria. Here the authors provide mechanistic insights into LPS recognition and transportation by determining the cryo-EM structures of the inner membrane complex LptB_2_FGC bound to either LPS or AMP-PNP.

## Introduction

Antibiotic resistance of Gram-negative bacteria has become one of the greatest threats to global health^1^. The asymmetric outer membrane (OM) of Gram-negative bacteria has a crucial role in defending against extracellular cytotoxic molecules such as antibiotics^2^. Lipopolysaccharide (LPS) is the main component of the OM, and its importance is not only in maintaining the OM structure, shielding from harmful molecules but also inducing host inflammatory immune responses causing disease-like sepsis^3–5^. Compromised OM integrity reduces virulence of pathogenic bacterial species and increases their sensitivity to antimicrobial agents^6^.

**Fig. 1:**
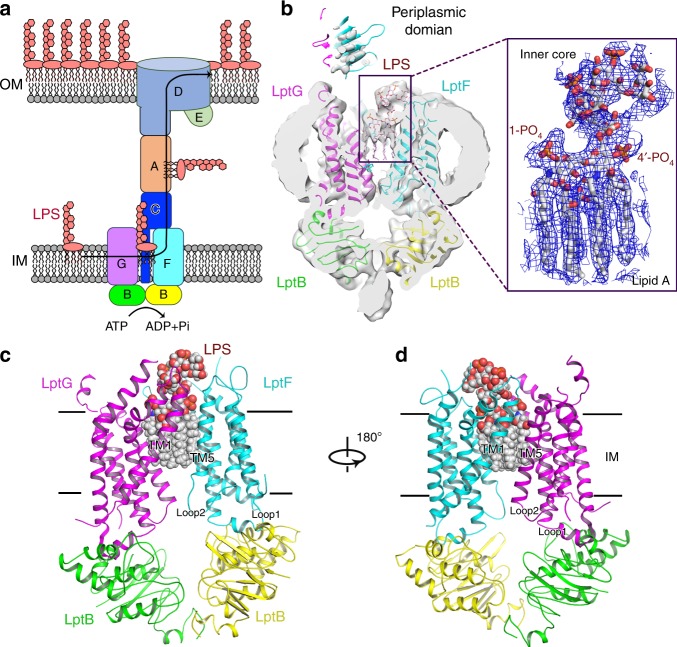
Architecture of LPS-bound *sf*LptB_2_FG complex. **a** A scheme of seven lipopolysaccharide transport proteins form a *trans*-envelope bridge to transport LPS from the IM to the OM. **b** Left panel: a cross-sectional view of cryo-EM map of LPS-bound *sf*LptB_2_FG; Right panel: a closed view of LPS. A clear density for LPS is shown in blue mesh and LPS is shown in stick. LptF, LptG and the two LptB molecules are presented in cartoon and coloured in cyan, purple, green and yellow, respectively. **c** Cartoon representation of LPS-bound *sf*LptB_2_FG. An LPS molecule, shown in spheres, is located in the upper cavity of the transmembrane channel. **d** Rotation of 180° along the *y*-axis relative to **c**

LPS is a large glycolipid consisting of lipid A, core oligosaccharide and O-antigen^5,7,8^. Components of LPS are synthesised in bacterial cytoplasm and then transported onto the periplasmic side of the inner membrane (IM), from where mature LPS is assembled and transported to the OM^9–12^. Seven LPS transport proteins (LptBFGCADE) form a *trans*-envelope bridge for LPS transport from the IM to the OM across the aqueous periplasm^13–25^ (Fig. 1a), which is a potential target for novel antimicrobial drugs^26–29^. The IM component LptB_2_FG comprises an ATP-binding cassette (ABC) transporter, which powers the transport of LPS across the bridge. The cytoplasmic LptB dimer binds and hydrolyses ATP and the transmembrane (TM) domains of LptF and LptG create a cavity to accommodate LPS (Fig. 1a). Unlike other canonical bacterial ABC transporters, which translocate substrate across the IM, LptB_2_FG acts by extracting LPS from the periplasmic side of the IM and delivering it to the periplasmic domain of the IM protein LptC^18,30–34^. LptC, which consists of a TM helix and a jellyroll-like periplasmic domain, forms a stable complex with LptB_2_FG to receive LPS and deliver it to the periplasmic protein LptA in the bridge^32,35^ (Fig. 1a).

To understand the mechanisms of how LptB_2_FGC recognises and acts to transport LPS, we obtained high-resolution cryo-electron microscopy (cryo-EM) structures of LptB_2_FGC complexed with substrate LPS or ATP analogue β-γ-imidoadenosine 5′-triphosphate (AMP-PNP). We also obtained cryo-EM structure of LptB_2_FG complexed with LPS to compare the conformational changes with and without the presence of LptC. Two recent studies reported LptB_2_FGC structures: the work of Owens et al.^36^ is an X-ray crystallographic study of *Vibrio cholerae* and *Enterobacter cloacae* LptB_2_FGC in detergent micelles and the work of Li et al.^37^ is a cryo-EM study of *Escherichia coli* LptB_2_FGC in lipid nano-discs with and without ADP-vanadate. Although LptB_2_FGC complex structures have been studied, due to partial occupancy or low resolutions in the substrate or nucleotide-binding pockets the two papers show no atomic evidence to determine LPS recognition in LptB_2_FGC complex or nucleotide-binding-induced transport mechanism^36,37^. By contrast, our high-resolution cryo-EM structures reveal atomic details in the LPS-binding and ATP-binding cavities, and mutagenic assays allowed us further to identify functional residues in the TM cavity and two periplasmic domains of LptF and LptG involved in LPS recognition and transport process including two essential arginine residues LptF R292 and LptG R301 in the cytoplasmic loop 2 of LptF or LptG. Conformational changes and molecular shifts between domains upon nucleotide binding reveal working mechanism of the transporter.

## Results

### The ATPase activities of purified LptB_2_FG and LptB_2_FGC

LptB_2_FG and LptB_2_FGC from *Shigella flexneri* (*sf*LptB_2_FG and *sf*LptB_2_FGC) were cloned, overexpressed, solubilised in *n*-dodecyl-β-d-maltopyranoside (DDM), and purified in Lauryl maltose-neopentyl glycol (LMNG) (Supplementary Fig. 1a, b). The ATPase activity of *sf*LptB_2_FG and *sf*LptB_2_FGC were measured (Supplementary Fig. 1c, d). Comparing to *sf*LptB_2_FG, the ATPase activity of *sf*LptB_2_FGC is about 50% less under the same conditions, confirming the reported regulatory role of LptC to the LptB_2_FG transporter^36,37^. The ATP non-hydrolysable analogue AMP-PNP significantly inhibits the ATPase activity of *sf*LptB_2_FGC, which was used to mimic and lock an ATP-bound state of *sf*LptB_2_FGC.

### Cryo-EM structure of LptB_2_FG in LPS-bound state

Our previous published crystal structure of LptB_2_FG from *Klebsiella pneumonia* (*kp*LptB_2_FG) did not identify the bound LPS molecule due to low resolution and low occupancy, although an extra electron density was found in the cavity^38^. In this study, we used cryo-EM to resolve an LPS-bound structure of *sf*LptB_2_FG at overall 3.7 Å resolution with TM domain at 3.2 Å resolution (Supplementary Figs. 2 and 3). The cryo-EM maps of *sf*LptB_2_FG show clear side chain densities in the nucleotide-binding domains (NBDs) and the two transmembrane domains (TMDs), allowing us to unambiguously fit models of NBDs and TMDs of LptB_2_FG (Fig. 1b). LptB_2_ constitutes the cytoplasmic NBD dimer and the six TM helices of LptF and LptG (F_TM1-TM6 and G_TM1-TM6) constitute two TMDs that are arranged to form a central cavity with two surface gaps between TM1F and TM5G and TM1G and TM5F termed as lateral gates (Fig. 1c, d). The densities for the periplasmic domains of LptF and LptG are at low resolution but are clearly visible (Fig. 1b and Supplementary Fig. 3). Each TMD has two cytoplasmic loops: loop 1 links the TM2 and TM3 helices through the coupling helix and loop 2 links the TM4 and TM5 helices of LptF and LptG (Fig. 1c, d).

**Fig. 2:**
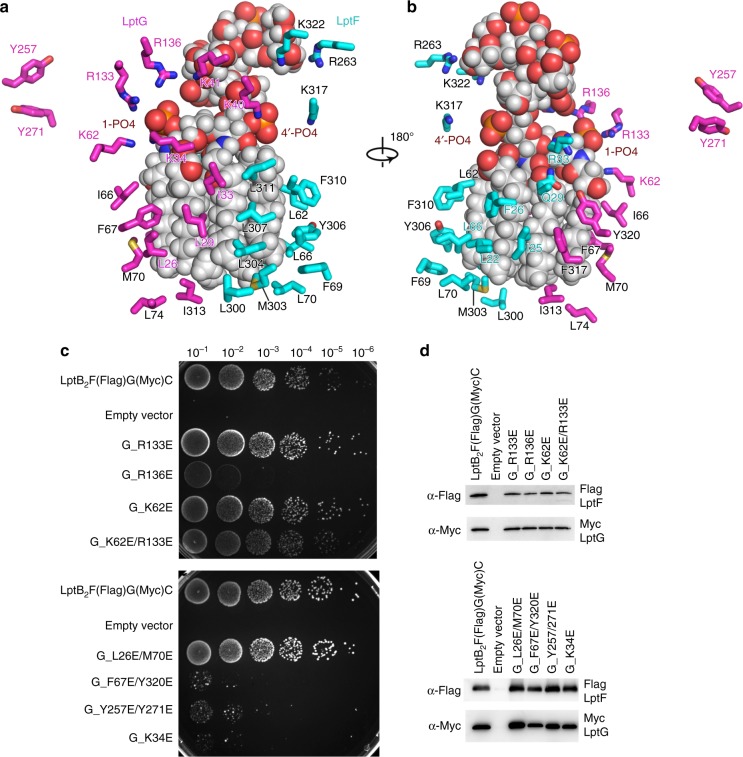
s*f*LptB_2_FG recognition of LPS. **a** Residues of LptF and LptG from the cavity that interact with LPS. LPS is shown in sphere and grey, whereas residues of LptF and LptG are shown in cyan and magenta, respectively. **b** 180° rotation of **a** along *y*-axis. **c** Functional assays of LptG residues. Mutants R136E, F67E/Y320E, Y257E/Y271E and K34E are lethal, whereas mutants R133E, K62E and L26E/M70E do not affect the bacterial growth. **d** Expression level of LptF and LptG of the mutants. The western blot showed that the mutant protein expression levels are similar to that of the wild type. Source data for panel **d** are provided as a Source Data file

The cryo-EM structure reveals a clear LPS density in the central cavity (Fig. 1b), showing all six acyl tails, glucosamine disaccharide phosphorylated at 1′ and 4′ positions, and the inner core oligosaccharide. The LPS molecule trapped is a natural substrate of *sf*LptB_2_FG that was overexpressed in the *E. coli* C43(DE3) strain. The acyl tails of LPS are drooped and perpendicular to the IM plane in the upper cavity and the inner core positioned above in the periplasmic space (Fig. 1b–d). In the cavity, hydrophobic residues I25, F26, L62, L66, L70 and M303 of LptF and L26, I33, I66, F67, I313, F317 and Y320 of LptG interact with the LPS acyl tails via van der Waals interactions. Charged residues K34, K62, R133 and R136 of LptG and R33 of LptF form salt bonds with the 1′-phosphate group of LPS, while K40 of LptG and K317 of LptF form salt bonds with the 4′-phosphate group of LPS. D37 of LptG interacts with the glucosamine disaccharide of lipid A. K322, R263 and Q248 of LptF and K41 of LptG interact with the inner core oligosaccharide (Fig. 2a, b). Previously, we reported that mutants of the hydrophobic residues F26D and L62D of LptF in the cavity severely impaired cell viability^38^. Here we carried out functional assay amongst those conserved charged and hydrophobic residues of LptG and found that K34E, R136E, R133E/K136E, Y257E/Y271E and F67E/Y320E are lethal (Fig. 2c, d and Supplementary Fig. 4). Interestingly, alanine substitution K34A and R136A and single mutant Y257A, Y271A, F67A and Y320A are normal (Supplementary Fig. 4). The positively charged K34 and R136 and the hydrophobic F67 and Y320 are located in the upper cavity in proximity to the negatively charged phosphate group and the hydrophobic acyl chains of the bound LPS, respectively. These results suggest that K34, R136, F67 and Y320 are important to LPS binding by forming ionic bonds and hydrophobic interactions, respectively, which can be compensated by each other if one of these interactions is lost as no effect was seen in the single alanine mutations. However, introducing negatively charged substitution would result repulsive force to destabilise the ionic bond thus affecting the functionality of the complex. On the other hand, Y257 and Y271 are located outside the cavity at the interface between the TM and the periplasmic domain of LptG (Fig. 2a–d and Supplementary Fig. 4). Although not interacting with LPS, double mutant Y257E/Y271E abolishes the functionality of the transporter causing cell death, suggesting that their roles are not in LPS recognition but may be involved in later stage of LPS transport to the bridge. In contrast, mutation of residues located in the lower cavity of LptG such as K13E/R86E showed no effect on cell viability, suggesting that the lower cavity is not involved in LPS binding (Supplementary Fig. 4). The high resolution within the LPS-binding cavity of the structure allowed us to visualise that these residues interact with the trapped LPS, supporting the results of the functional assays carried out here and reported before^39,40^.

### Cryo-EM structures of LptB_2_FGC in LPS-bound state

**Fig. 3:**
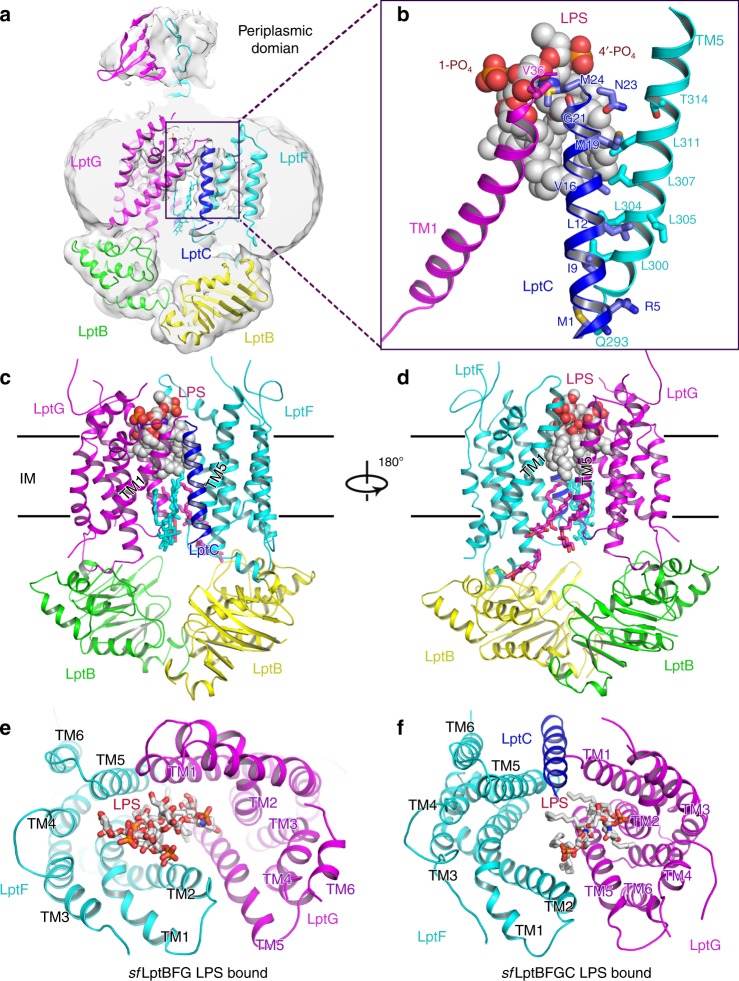
Cryo-EM structure of LPS-bound *sf*LptB_2_FGC. **a** Cryo-EM map of LPS-bound *sf*LptB_2_FGC. The colour scheme is the same as in Fig. 1. LptC is coloured in blue. **b** A close view of LPS. Lipid A of LPS is visible in the complex and LptC transmembrane helix is at the lateral gate TM1G/TM5F. LPS is shown in spheres. **c** Cartoon representation of LPS-bound *sf*LptB_2_FGC. **d** Rotation of 180° along the *y*-axis relative to **c**. **e** Periplasmic view of LPS-bound *sf*LptB_2_FG, where LPS is closed to the LptF side. **f** Periplasmic view of LPS-bound sfLptB_2_FGC. LPS is closed to the LptG side

The cryo-EM structure of *sf*LptB_2_FGC was determined to 3.1 Å resolution (Fig. 3a–d and Supplementary Figs. 5 and 6). The map shows clear densities of the TM helix of LptC located at one lateral gate between TM1G and TM5F, which is consistent to the published structures^36,37^ (Fig. 3a and Supplementary Fig. 6). However, due to possible flexibility, we are unable to see clear density for the periplasmic domain of LptC (Fig. 3a and Supplementary Fig. 6). This was also the case in the recent reported cryo-EM structure of LptB_2_FGC, where most of particles collected do not show density for the periplasmic domain of LptC^37^, whereas on the other hand the reported crystal structure of LptB_2_FGC was able to show clear periplasmic domains^36^. In our structure, residues M1, R5, I9, L12, V16, M19 and N23 from the TM helix of LptC interact with residues Q293, L300, L305, L304, L307, L311 and T314 from the TM5 of LptF, respectively. In contrast, only G21 from the TM helix of LptC interacts with V36 of TM1 of LptG (Fig. 3b). Comparing to the *sf*LptB_2_FG LPS-bound structure, the presence of the TM helix of LptC makes the lateral gate TM1G/TM5F of *sf*LptB_2_FGC structure much widely opened, along with the neighbouring TM2 and TM3 of LptG and TM4 and TM6 of LptF moved outward, resulting an enlarged central cavity (Fig. 3e, f). In contrast to the two recent publications^36,37^, a density for LPS is identified in the cavity of *sf*LptB_2_FGC with all features visible as in the *sf*LptB_2_FG structure except the inner core of the LPS (Fig. 3a–d). Interestingly, the LPS molecule trapped in the cavity of *sf*LptB_2_FGC is about 7.3 Å away from that of *sf*LptB_2_FG structure referring to the position of the 1′-phosphate group (Fig. 3e, f). The LPS molecule in the *sf*LptB_2_FGC structure positions in proximity to LptG with kinked acyl tails rather than drooped as seen in the *sf*LptB_2_FG structure (Fig. 3b–d and Supplementary Fig. 6). In the cavity of *sf*LptB_2_FGC, we can also see that hydrophobic residues L17 and M24 from the TM helix of LptC interact with the bound LPS (Supplementary Fig. 7), suggesting that the TM helix of LptC is also involved in LPS recognition. The number of residues from the TM helices of LptF and LptG that have been shown to interact with LPS in the enlarged cavity of *sf*LptB_2_FGC are greatly reduced compared to the structure of *sf*LptB_2_FG (Fig. 3a, b and Supplementary Fig. 7). The weaker LPS binding in the cavity of *sf*LptB_2_FGC suggests an energy regulatory role of LptC in the transporter. The presence of LptC in the structure enlarges the gap at one lateral gate TM1G/TM5F, which makes one to speculate that LPS may enter the cavity via this lateral gate. We can visualise a detergent molecule (LMNG) trapped at this lateral gate. However, we found one acyl chain of the trapped LPS stuck at the opposite lateral gate TM1F/TM5G (Supplementary Fig. 8), suggesting that hydrophobic molecules to enter through these lateral gates is also possible.

### Cryo-EM structures of LptB_2_FGC in AMP-PNP-bound state

**Fig. 4:**
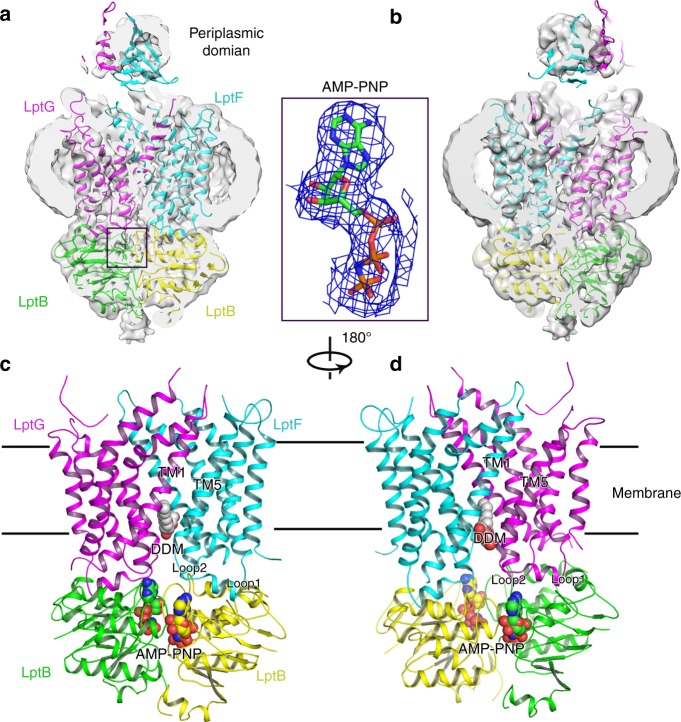
Structure of AMP-PNP-bound *sf*LptB_2_FGC. **a** Cryo-EM map of AMP-PNP-bound *sf*LptB_2_FGC. The LptC density is not observed. Density of AMP-PNP is shown in blue mesh and AMP-PNP is shown in stick. The colour scheme is the same as Fig. 1. **b** Rotation of 180° along the *y*-axis relative to **a**. **c** Cartoon representation of AMP-PNP-bound *sf*LptB_2_FGC. AMP-PNP is shown in spheres with carbon colour in green or yellow. DDM is shown in sphere with carbon in grey. **d** Rotation of 180° along *y*-axis relative to **c**. The DDM molecule is trapped in the lateral gate TM1F/TM5G

**Fig. 5:**
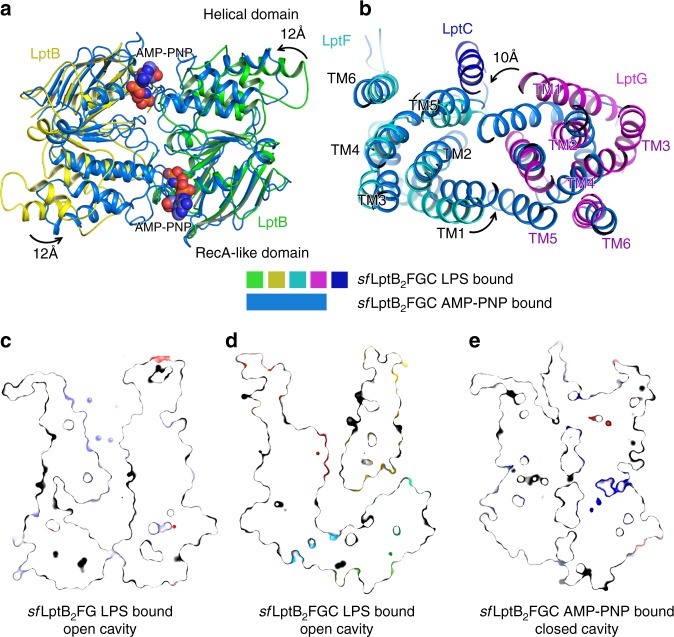
Superimpositions of AMP-PNP and LPS-bound *sf*LptB_2_FGC structures. **a** AMP-PNP binding induces the conformational changes of LptB (NDBs). The helical domains of the dimeric LptB (AMP-PNP-bound *sf*LptB_2_FGC) showed a rotational shift of ~12 Å in the anti-clockwise direction from the nucleotide-free state of dimeric LptB (LPS-bound *sf*LptB_2_FGC). The LPS-bound *sf*LptB_2_FGC is coloured in the same as Fig. 1. The AMP-PNP-bound *sf*LptB_2_FGC is coloured in marine blue and AMP-PNP shows as spheres. **b** A top view of superimposed transmembrane helices of LPS-bound *sf*LptB_2_FGC and AMP-PNP-bound *sf*LptB_2_FGC complex. AMP-PNP binding induces the TM helices of AMP-PNP-bound *sf*LptB_2_FGC to rotate in the anti-clockwise direction with the largest shift of ~10 Å from the nucleotide-free state (LPS-bound *sf*LptB_2_FGC) towards the central channel. **c** Slab view of surface representation of cavity of LPS-bound *sf*LptB_2_FG. The cavity is in an outward open conformation. **d** Slab view of cavity of LPS-bound *sf*LptB_2_FGC. The cavity is in an outward open conformation. The transmembrane helix of LptC is located at the lateral gate TM1G/TM5F, enlarging the cavity. The neck of the cavity is widely open. **e** Slab view of cavity of AMP-PNP-bound *sf*LptB_2_FGC. The cavity is closed, which is induced by AMP-PNP binding

To obtain a nucleotide-bound structure, we incubated the purified protein *sf*LptB_2_FGC with the non-hydrolysable ATP analogue AMP-PNP. Unlike the *sf*LptB_2_FGC LPS-bound structure, LptC is moved away from the lateral gate TM1G/TM5F so that the TM helix became invisible in the complex structure despite of the high resolution of 3.5 Å showing near atomic details in the cavity of the transporter (Fig. 4a–d, and Supplementary Figs. 9 and 10). Densities for the periplasmic domains of LptF and LptG are observed at low resolution but the periplasmic domain of LptC is unable to be visualised, which is also the case in the recently reported ADP-vanadate complexed LptB_2_FGC structure^37^ (Supplementary Fig. 10). Superimposition of the two cryo-EM structures of *sf*LptB_2_FGC LPS-bound and *sf*LptB_2_FGC AMP-PNP-bound showed two different conformations with a root mean square deviation (RMSD) of 3.21 Å over 589 aligned Cα atoms (Fig. 5a, b), representing two different states of the transporter.

In our structures, the *sf*LptB_2_FG and *sf*LptB_2_FGC process an open cavity to accommodate LPS (Fig. 5c, d), while the *sf*LptB_2_FGC AMP-PNP-bound structure adopts a closed central cavity with a closed dimeric conformation of LptB (Fig. 5e). The NBDs (LptB_2_) of our *sf*LptB_2_FGC AMP-PNP-bound structure is similar to that of the ATP-bound *E. coli* LptB dimer (PBD: 4QC2) with a RMSD of 0.91 Å over 457 aligned Cα atoms^41^, particularly their bound nucleotides and the binding elements of LptB match well (Supplementary Fig. 11). A superimposition of the *sf*LptB_2_FGC AMP-PNP-bound structure and the nucleotide-free *sf*LptB_2_FGC LPS-bound structure shows that the binding of AMP-PNP causes an anti-clockwise rotation in the C terminal helical domains of LptB_2_ towards the dimerisation interface (Fig. 5a). The AMP-PNP binding also induces the conformational changes of TM1–5 of LptF and TM1–5 of LptG to rotate anti-clockwise toward the centre of the cavity (Fig. 5b), resulting in a closed central cavity with a widely opened neck at the periplasmic side of the cavity (Fig. 5e). No densities of LPS in this closed cavity was observed, suggesting a post-exporting state of LPS. Two lateral gates TM1F/TM5G and TM1G/TM5F shift into a closed conformation with their respective TM1 and TM5 helices oriented parallel to each other in close proximity, a ‘*cis*’ conformation (Supplementary Fig. 8). In this structure, a detergent molecule (DDM) is trapped at the lateral gate TM1F/TM5G, with hydrophobic tails trapped in the cavity while its hydrophilic head extends into the periplasm. The hydrophobic residues L16, I18, I21, I25 and M74 of LptF, and V309, V310, I313, L74, L78 and M25 of LptG in the lumen of the cavity interact with the detergent molecule (Supplementary Fig. 8). Some of these hydrophobic residues (e.g. residues I25 of LptF, residues L74 and I313 of LptG) are also involved in LPS binding (Fig. 2a, b). As a result, the state of the trapped detergent molecule in the lateral gate may suggest the way that LPS enters the cavity of *sf*LptB_2_FGC.

### LptF R292 and LptG R301 interact with the bound AMP-PNP

**Fig. 6:**
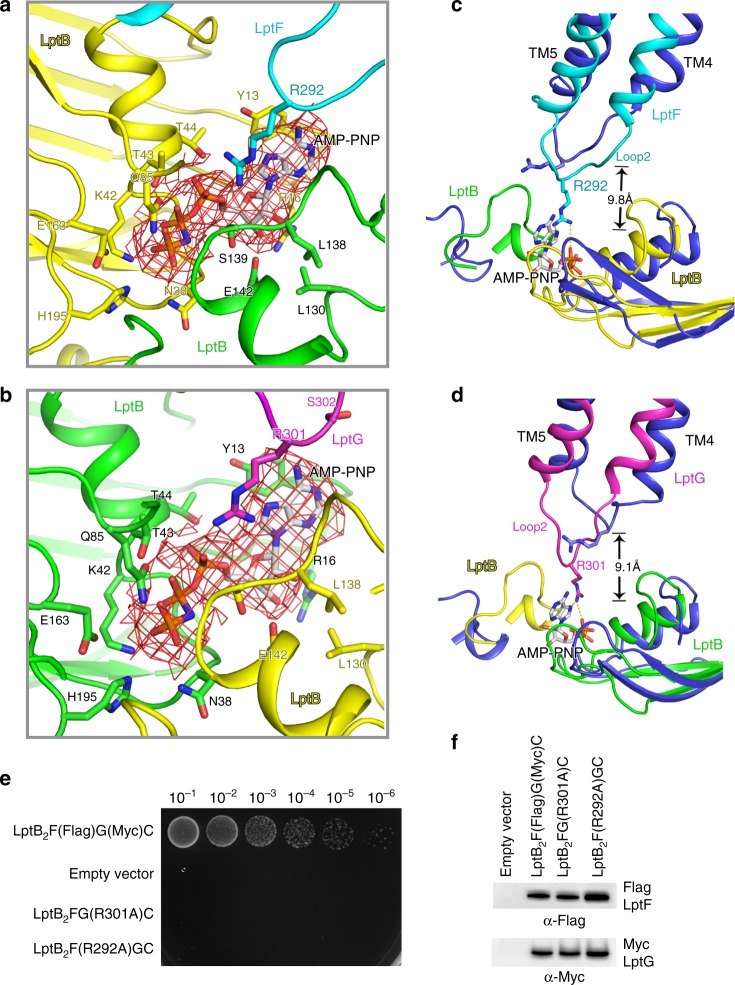
R292 of LptF and R301 of LptG are involved in AMP-PNP binding. **a** A close view of AMP-PNP binding residues with dimeric LptB and R292 of LptF. **b** A close view of AMP-PNP binding residues with dimeric LptB and R301 of LptG. AMP-PNP molecules are shown in stick, and the cryo-EM map for AMP-PNP are shown in red mesh. **c** The two cryo-EM structures are superimposed. The arginine residue R292 located on the cytoplasmic loop 2 of LptF shifts around 9.8 Å to interact with AMP-PNP. **d** The arginine R301 located on the cytoplasmic loop 2 of LptG shifts around 9.1 Å to interact with the AMP-PNP. AMP-PNP molecules are shown in stick, the colour scheme of *sf*LptB_2_FGC AMP-PNP bound is the same as in Fig. 4, and *sf*LptB_2_FG LPS bound is coloured in blue. **e** Functional assays of the single mutants R292 of LptF and R301 of LptG. NR1113 cells were transformed with empty vector (pTRC99a_Kan, the negative control) or the vector encoding LptB_2_F(Flag)G(Myc)C (the positive control). **f** Detection of protein expression levels of mutants by western blotting. Empty vector (pTRC99a_Kan, the negative control) or the vector encoding LptB_2_F(Flag)G(Myc) (the positive control). The bacterial cells for western blotting were cultured in the presence of 0.2% l-arabinose. All results have been confirmed at least three times. Source data are provided as a Source Data file 6**f**

The *sf*LptB_2_FGC AMP-PNP-bound structure shows a closed dimeric conformation of LptB with clear densities for two bound AMP-PNP molecules in the ATP-binding sites at the interface of the NBDs (LptB dimer) (Fig. 4a–d). Several residues are involved in the AMP-PNP binding site, including Y13, T44, T43, Q85, K42, E163, H195 and N38 from one LptB of the NBDs and E142, L138 and S139 from another LptB of the NBDs (Fig. 6a, b). Some of these residues have been tested by the functional assays in previously published studies showing that mutations of these residues impaired bacterial cell viability^31,41^. In addition to the residues of LptB_2_, we also found that two arginine residues LptF_R292 and LptG_R301 on the cytoplasmic loop 2 of LptF and LptG extended ~9.8 and ~9.1 Å, respectively, toward the NBDs to interact with the bound AMP-PNP molecules (Fig. 6c, d). To test whether these arginine residues are critical for the functionality of *sf*LptB_2_FGC, we made two single mutants LptF R292A and LptG R301A and tested cell viability and ATPase activity of the transporter. Interestingly these mutations showed no impact on the ATPase activity of the complex (Supplementary Fig. 1) but caused cell death in the functional assays (Fig. 6e, f), suggesting that LptF R292 and LptG R301 residues are essential for LPS transporting but not ATP hydrolysing function of the transporter. As these loops connect TM helices that constitute the central cavity and lateral gates, we speculate that these cytoplasmic R292 and R301 containing loops probably act similarly to the coupling helices (cytoplasmic loop 1), which trigger conformational changes in the TMDs by allowing allosteric communication between the NBDs and TMDs of LptB_2_FGC upon nucleotide binding^33^.

### Periplasmic domains of LptF and LptG may transport LPS

**Fig. 7:**
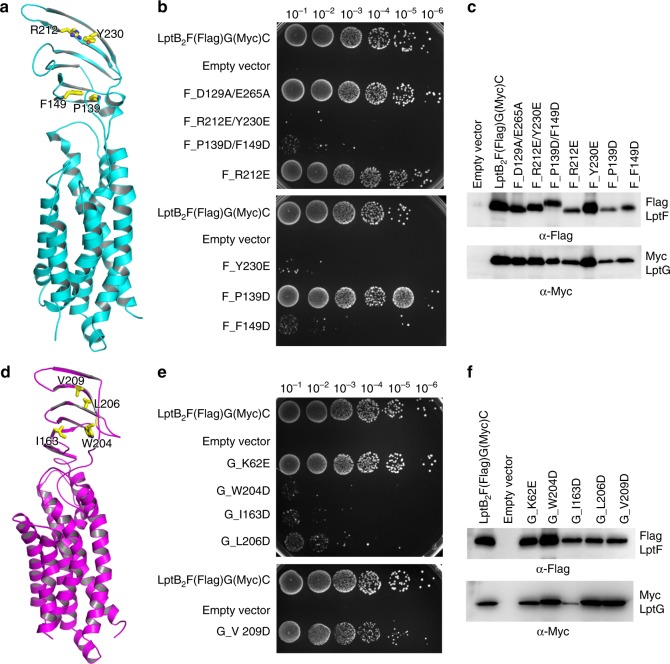
Function of periplasmic domains of LptF and LptG. The structure of the β-jellyroll-like periplasmic domains of LptF and LptG is similar to that of LptC. **a** Cartoon representation of LptF. Hydrophobic β-jellyroll-like core residues F149, P139, Y230 and R212 are shown in stick. **b** Functional assays of LptF residues. Residues D129 and E265 are at the neck of the cavity as a control. Mutants R212E/Y230E, P139D/F149D, Y230E and F149D are lethal, while mutants D129A/E265A, R212E and P139D do not have any impact on cell growth. **c** Protein expression level of the mutants was detected by western blotting. Protein expression levels of other mutants are higher than that of P139D. Source data are provided as a Source Data file (7**c**). **d** Cartoon representation of LptG. Hydrophobic β-jellyroll-like core residues I163, W204, L206 and V209 are shown in stick. **e** Functional assays of LptG residues. K62 is located at the cavity as a control, and the mutant K62E grows the same as the wild type. Mutants W204D, I163D, and L206D are lethal, and mutant V209D reduces the cell growth. **f** Protein expression level of mutants was detected by western blotting. Protein expression level of other mutants is higher than that of mutant K62E except mutant I163D. The lethality of mutant I163D might cause by the lower protein expression. Source data are provided as a Source Data file (7**f**)

Our work has showed that both LptF and LptG residues in the cavity of the transporter *sf*LptB_2_FGC are essential and hydrophobic molecules like detergents can enter via both lateral gates (Supplementary Fig. 8). We wondered whether both periplasmic domains of LptF and LptG are crucial for the functionality of the transporter. To test this, we generated single or double mutants located in the core of β-jellyroll-like periplasmic domains of LptF or LptG and performed functional assays. Mutants of conserved residues of W204D, I163D and L206D in LptG and R212E/Y230E, P139D/F149D, Y230E and F149D in LptF are lethal or severely impair cell growth (Fig. 7a–f). The lethality of I163D may be due to the lowered protein expression compared to that of the wild type. Nevertheless, both periplasmic domains contain functional residues that are crucial for LptB_2_FGC, which might suggest that both of the periplasmic domains of LptF and LptG are involved in LPS transport.

## Discussion

The three cryo-EM structures have revealed different conformations of transporter and distinct configurations of the bound LPS in the presence and absence of LptC, which allowed us to speculate the function of LptC in the transporter. In the *sf*LptB_2_FGC LPS-bound structure, the TM helix of LptC located between TM1G and TM5F opens up the lateral gate and enlarges the central cavity (Fig. 3a–d). Residues from TM helices of all LptC, LptG and LptF in the cavity make interactions with the bound LPS suggesting all three protein components are involved in LPS binding (Supplementary Fig. 7). However, both *sf*LptB_2_FG and *sf*LptB_2_FGC structures trapped LPS, suggesting that extracting LPS from the IM into the cavity is not determined by the presence of LptC but LptC is present to facilitate the entry of LPS and direct its transport toward the bridge. Deletion of the full length LptC greatly diminishes LPS transport to LptA^42^, suggesting its importance in bridging the IM transporter and periplasmic protein LptA. Moreover, the enlarged cavity in the *sf*LptB_2_FGC structure resulted in weaker LPS binding in the cavity with reduced interactions between LPS and cavity residues compared to *sf*LptB_2_FG (Fig. 2a, b and Supplementary Fig. 7), implicating the regulatory role of LptC in making LPS transport more energy efficient. This is also supported by the reduced ATPase activity of *sf*LptB_2_FGC compared to *sf*LptB_2_FG under the same conditions (Supplementary Fig. 1). The TM helix of LptC opens up the lateral gate TM1G and TM5F, makes a possible entrance for LPS into the cavity supporting the reported models^36,37^ (Supplementary Figs. 12 and 13). On the other hand, one of the LPS acyl tails and an amphipathic detergent molecule interact at the opposite lateral gate (TM1F/TM5G) of the structures, which may suggest that this lateral gate TM1F/TM5G could also be accessible by LPS or amphipathic molecules in cells (Supplementary Fig. 8).

In the *sf*LptB_2_FGC structure, LptF makes most of interactions with LptC, and LptG only interacts with LptC through one residue. Two recently reported structures show that the periplasmic domain of LptC connects to that of LptF^36,37^, this makes one to recognise the importance of LptF in LPS transport (Supplementary Fig. 13). On the other hand, our mutagenic studies reveal that the residues of LptG both in the TM cavity and periplasmic domain show remarkable functional importance to cell viability. Moreover, the trapped LPS molecule in the *sf*LptB_2_FGC structure shifted 7.3 Å toward LptG compared with the LPS in *sf*LptB_2_FG structure (Fig. 3e, f). As a result, we speculate that LptG also has important roles in LPS recognition and transportation. Further investigations are required to clarify the exact path of LPS transported within the transporter system.

In the *sf*LptB_2_FGC AMP-PNP-bound structure, the TM helix of LptC moves away from the LPS-binding cavity. As a result, the TM helices of LptF and LptG may be able to move freely to change conformation to expel LPS out of the cavity. A comparison of the structures shown here provides insight into the transport cycle of *sf*LptB_2_FGC. In the nucleotide-free state, both the NBDs and TMDs are in open conformations. An LPS in the outer leaflet of the IM binds laterally into the central channel of *sf*LptB_2_FG through an open lateral gate (Figs. 1c, d and 5c and Supplementary Fig. 8). The opening of lateral gates in this state exposes LPS-binding elements in the lumen of the TM channel. Interactions of the hydrophobic and charged residues of the TM helices with the six fatty acyl chains and phosphate groups of LPS may provide sufficient strength and specificity for LPS extraction (Fig. 2a, b).

Upon nucleotides binding, two NBDs dimerise into a closed conformation (Fig. 5a), triggering conformational changes in the TMDs via the coupling helices and probably the cytoplasmic loop 2. The conformational changes in the NBDs induce an anti-clockwise rotation of the TM helices of LptF and LptG towards the centre to close the cavity (Fig. 5e and Supplementary Movies 1 and 2), like the closing motion of a camera lens aperture. The two arginine residues (R292 of LptF and R301 of LptG) identified interacting with the bound nucleotides may have roles in switching the states of the lateral gates of *sf*LptB_2_FGC as these residues locate on the loop 2 that links respective TM5 of LptF and LptG composing the lateral gates. When comparing the ‘*trans*’ and ‘*cis*’ conformations of TM1 and TM5 of the lateral gates at the two states (Supplementary Fig. 8), it is apparent that the gates are in closed states when the complex binds to AMP-PNP (Fig. 5c, d).

The induced inward rotation of the TM helices results tightened TM cavity (Fig. 5a, b), squeezing the bound LPS into the periplasmic domain of LptB_2_FGC. The structure of the periplasmic domains of LptF and LptG are similar to that of LptC^38,39^, and the periplasmic protein LptA shares a common fold with LptC^16,18,35,43–45^ and the N terminal domain of LptD^21,22^. Together they form a head-to-tail oligomer with a continuous whirling orientated hydrophobic groove bridging between the IM and the OM^25,30,46–48^. The rotational motion of LptB_2_FGC observed in our structures agrees with the models proposed before^11,25,46^ that the rotating action from the base brings the bound LPS into the spiralling bridge. The design of the rotational mechanism and the need of LptC allows efficient transport of LPS through membrane bridge powered only by LptB_2_FG. The non-hydrolysable nucleotide AMP-PNP binding is able to induce conformational change suggesting that LPS expelling does not require ATP hydrolysis. The arginine residues on the loop 2 interact with the first phosphate group of AMP-PNP suggesting that the conformation of the transporter will only return to the original ‘open’ state when the nucleotide is released after hydrolysis.

Our cryo-EM structures of *sf*LptB_2_FGC show similarities in the TM domains and nucleotide-binding domains of the transporter when superimposed with previously published cryo-EM or crystal structures (Supplementary Fig. 13). The periplasmic domains of LptF and LptG are however shown at different conformations, revealing their flexibility. These data suggest that the structure of LptB_2_FGC is highly conserved across species, which makes it an important protein machinery complex to study for understanding the biosynthesis of Gram-negative bacterial membrane.

In summary, our cryo-EM structures reveal two important intermediate states of *sf*LptB_2_FGC at high resolution, providing molecular basis for substrate recognition and ATP induced conformational change of LptB_2_FGC. The comparison of the conformations provides a full picture of the transporting mechanism of LptB_2_FGC at the NBDs and TMDs. This finding confirms rotational transporting model and explains how energy efficiency is achieved by the ABC transporter in the presence of LptC.

## Methods

### Expression and purification of LptB_2_FGC and LptB_2_FG

Gene fragments containing *lptB* and *lptF-lptG* of *S. flexneri* strain were amplified separately by PCR and subsequently cloned into a pTRC99a plasmid (*Eco*RI/*Kpn*I restriction digestion for *lptB* and *Kpn*I/*Xba*I digestion for *lptF-lptG*), resulting a pTRC99a- *lptB*_*2*_*FG* construct with an octa-histidine (8 × His) tag at the C terminus of LptB. Primers are listed in Supplementary Table 2. The resulting plasmid was transformed into *E. coli C43 (DE3)* cells (Novagen) for protein expression. The bacterial cells were grown in Luria broth (LB) supplemented with antibiotic (100 µg ml^−1^ ampicillin) at 37 °C until the optical density of the culture reached 0.6 at a wavelength of 600 nm (OD_600_). LptB_2_FG expression was induced with 0.1 mM isopropyl-β-d-thiogalactopyranoside (IPTG) at 20 °C for 6 h. The *lptC* gene was amplified from *S. flexneri* strain and the plasmid pTRC99a- *lptB*_*2*_*FG* was linearised by PCR individually. Subsequently, the fragments of *lptC* and pTRC99a-*lptB*_*2*_*FG* were ligated to create pTRC99a-*lptB*_*2*_*FGC*. *C43 (DE3)* cells transformed with pTRC99a- *lptB*_2_*FGC* were cultured and induced at the same conditions as described above for over-expression of LptB_2_FG.

Cultures were harvested by centrifugation and cell pellets were resuspended in purification buffer (20 mM HEPES pH 7.8 and 300 mM NaCl) supplemented with 0.1 mM phenylmethylsulphonyl fluoride (PMSF, Sigma-Aldrich). The cells were lysed by three passes through a cell disrupter (ATS Engineering Ltd) and cell debris was removed by centrifugation at 18,000 × *g* for 15 min at 4 °C. Membranes were pelleted by ultracentrifugation at 100,000 × *g* for 1 h at 4 °C and solubilized in purification buffer supplemented with 10 mM imidazole and 1% (w/v) *n*-dodecyl-β-d-maltopyranoside (DDM) (Anatrace) by stirring at room temperature for 1 h. The suspension of solubilized protein was ultracentrifuged at 100,000 × *g* for 1 h before being loaded onto a 5 ml HisTrap HP column (GE HealthCare). The column was washed with purification buffer supplemented with 0.05% Lauryl Maltose Neopentyl Glycol (LMNG) and 50 mM imidazole and bound protein was eluted with purification buffer supplemented with 0.05% LMNG and 300 mM imidazole. The protein eluted from Histrap HP column was further purified by size-exclusion chromatography using a Superdex 200 Increase 10/300 column (GE Healthcare) equilibrated in 20 mM HEPES, pH 7.8, 150 mM NaCl and 0.05% LMNG. The purities of the protein fractions were analysed by SDS–PAGE. Fractions with highest purity were collected and concentrated for cryo-sample preparation. For *sf*LptB_2_FGC AMP-PNP bound, the purified *sf*LptB_2_FGC was incubated with 5 mM β-γ-imidoadenosine 5′-phosphate (AMP-PNP) and 2 mM MgCl_2_ for 1 h at room temperature before cryo-sample preparation.

### ATPase activity assay

ATPase activity assay was performed using ATPase/GTPase Activity Assay Kit (Bioassay Systems). *C43 (DE3)* cells carrying *sf*LptB_2_FGC, *sf*LptB_2_FG or their mutated plasmids were cultured in 1 l LB medium. Cells were induced, collected and lysed using the protocol described above. Solubilised membrane fraction was ultracentrifuged at 100,000 × *g* for 30 min and the supernatants of each sample were loaded onto a gravity column containing 2 ml pre-balanced Ni^2+^-NTA beads. The columns were washed with 15 column volumes of wash buffer (20 mM HEPES pH 7.8, 300 mM NaCl, 50 mM imidazole and 0.05% LMNG), and eluted with the elution buffer (20 mM HEPES pH 7.8, 300 mM NaCl, 300 mM imidazole and 0.05% LMNG). All samples were further purified using size-exclusion chromatography with a Superdex 200 Increase 10/300 column (GE Healthcare).

Protein concentration of the samples was determined using detergent compatible Pierce BCA Protein Assay Kit (Thermo Scientific) according to the manufacture’s instruction. Briefly, 2.5 μl of purified protein was diluted to 25 μl for the BCA assay. The albumin (BSA) was used as the standard. An aliquot of 200 μl of working reagent (made by mixing reagent A and reagent B at 50:1 volume ratio) was added to each sample and incubated at 37 °C for 30 min. Absorbance at 562 nm was measured and the protein concentration of each sample was determined.

The ATPase activity assays were carried out in 96-well plates. The phosphate standards and blank control for colorimetric detection was prepared according to the manufacturer’s instructions (ATPase/GTPase Assay Kit, Bioassay systems). An aliquot of 1 μl (1–2 mg ml^−1^) of samples was mixed with 4 μl detergent buffer (20 mM HEPES, pH 7.8, 150 mM NaCl and 0.05% LMNG) and 5 μl assay buffer (ATPase/GTPase Assay Kit) to make 10 μl ATPase activity assay sample. 30 μl reaction solution (made by 20 μl assay buffer plus 10 μl 4 mM ATP solution) was added into each ATPase activity assay sample and incubated at room temperature for 15 min. The reaction was terminated by adding 200 μl reagent (ATPase/GTPase Assay Kit) into each sample and further incubated for 30 min. The absorbance at 600 nm was measured. For AMP-PNP inhibition, *sf*LptB_2_FGC was incubated with 2 mM AMP-PNP and 5 mM MgCl_2_ at 37 °C for 30 min before ATPase activity assay was performed. All assays were repeated six times. ATPase activities of all samples were determined using the mean value of the samples according to the linear regression of standards. All experiments were repeated at least three times.

### Site-directed mutagenesis and functional assays

All single or double mutations were generated following the site-directed mutagenesis protocol published by Liu and Naismith^49^. The functional assays were conducted on the *E. coli lptFG* chromosomal deletion strain NR1113 (Courtesy to N. Ruiz.)^13^. *lptFG* deletions are lethal, therefore NR1113 carries a rescue copy of *lptFG* operon with ampicillin resistance and P_BAD_ promotor, which is inducible by arabinose. The pTRC99a plasmid’s ampicillin resistance gene was replaced by a kanamycin resistance gene, which was then used as the vector for the *E. coli* LptB_2_FGC mutagenesis. In addition to the His ×8 tag at the C terminus of LptB, we also inserted a Flag tag at residue 138 of LptF (LptF-138-Flag) and a c-Myc tag at 144 of LptG (LptG-144-Myc) to generate the resulting plasmid pTRC99a-*E. coli LptBF138G144C*-Kan.

These single or double mutants were transformed into the *E. coli lptFG* deletion strain NR1113^13^. The transformed *E. coli* cells were grown on LB agar plate supplemented with antibiotics (kanamycin 50 µg ml^−1^) and 0.2% l-arabinose at 37 °C for 12 h. Single colonies of each transformation were inoculated into 10 ml LB medium supplemented with the antibiotics and 0.2% (w/v) l-arabinose. The cells were cultured in an incubator at 200 r.p.m. and at 37 °C for 12 h. Subcultured cells were used for the functional assays. The *E. coli* NR1113 with the empty plasmid pTRC99a-Kan was used as the negative control, while the NR1113 strain with the plasmid pTRC99a-Kan-LptBF(Flag)G(Myc)C or the NR1113 strain in the presence of 0.2% l-arabinose was used as the positive control. Cell pellets were harvested, washed twice and diluted in sterile LB medium to the OD_600_ nm of 0.5. Tenfold serial dilution functional assays were performed. The dilution range was from 10^−1^ to 10^−6^ and 5 µl of the diluted cells was dripped onto the LB agar plates containing kanamycin 50 μg ml^−1^. Cell growth was observed after overnight culture at 37 °C. All the assays were performed in triplicate.

### Western blotting

The protein expression of the LptFG mutants were determined by western blotting. An aliquot 0.5 ml of overnight cultures of transformed NR1113 cells with LptB_2_FGC or mutants was inoculated into 50 ml LB supplemented with antibiotics (kanamycin 50 µg ml^−1^) and 0.2% l-arabinose. The cells were cultured at 37 °C for 6 h and harvested by centrifugation. The cells were resuspended in 1 ml buffer containing 20 mM Tris-Cl, pH 7.8 and 150 mM NaCl supplemented with 1 mM PMSF. The cells were lysed by sonication for 1 min on ice. The membrane fraction was harvested and solubilized with 1% DDM for 20 min at room temperature. The undissolved debris was removed by centrifugation at 13,000 × *g* for 10 min at 4 °C. The supernatant was loaded to a Ni^2+^-NTA column and washed with a buffer containing 0.05% DDM, 20 mM Tris-Cl pH 7.8, 150 mM NaCl and 30 mM imidazole. The protein was eluted with 0.05% DDM, 20 mM Tris-Cl pH 7.8, 150 mM NaCl and 500 mM imidazole. The eluted samples were mixed with 4 × SDS–PAGE loading buffer and incubated at 98 °C for 10 min. The samples were centrifuged at 13,000 × *g* for 1 min, and 10 μl of each sample was loaded onto 12% Bis-Tris Plus SDS–PAGE gel for the immunoblot analysis.

The proteins were transferred to a PVDF membrane using the Mini Transfer-Blot (Bio-Rad) at 100 V for 1 h. The PVDF membranes were blocked in 1× phosphate buffered saline Tween-20 (PBST) supplemented with 5% skim milk at 4 °C for 1 h. The membranes were incubated with anti-Flag (Sigma, Catalogue No: F3165) or anti-Myc monoclonal antibody (1:300 dilution) (Sigma, Catalogue No: A5963) at room temperature for 1 h. The membranes were washed with PBST four times and then incubated with rabbit anti-mouse IgG antibody (1:5000 dilution) for 1 h. The membranes were washed with PBST four times and PBS twice. Protein bands were visualised by chemiluminescent in a photo imager (Bio-Rad).

### Sample preparation and cryo-EM data acquisition

2.5 μl of purified protein complex at a concentration of ~1 mg ml^−1^ was applied to glow-discharged Quantifoil holey carbon grids (R1.2/1.3, 300 mesh, copper). Grids were blotted for 3.5 s with the environmental chamber set at 95% humidity and flash-frozen in liquid ethane cooled by liquid nitrogen using Vitrobot Mark IV (FEI). Grids were imaged with a Titan Krios (FEI) electron microscope, operated at 300 keV equipped with a K2 Summit electron counting direct detection camera (Gatan). Datasets were collected in super-resolution mode using the automated data collection programme SerialEM^50^. All cryo-EM images were recorded in super-resolution mode and images were acquired at a nominal magnification of ×29,000, corresponding to a calibrated physical pixel size of 1.014 Å. Defocus range was set between −1.5 and −2.5 μm. Each image was acquired at an exposure time of 8 s and dose-fractionated to 40 frames with a dose rate of about 7 counts per second per physical pixel.

### Image processing

For the cryo-EM data of *sf*LptB_2_FG LPS bound, the beam-induced motion correction of image stacks were performed using MotionCor2 to generate 2x binned average micrographs and dose-weighted micrographs with a pixel size of 1.014 Å^51^. The contrast transfer function parameters of these average micrographs were estimated by Gctf^52^. Other procedures of data processing were performed in RELION^53^. 1,524,391 particles were automatically selected, and finally 95,887 particles were selected for 3D refinement and a reported 3.7 Å resolution map was generated after post-processing with a *B*-factor of −107 Å^2^. The data processing details are summarised in Supplementary Fig. 2.

The data processing procedures of *sf*LptB_2_FGC LPS bound were similar to the procedures of *sf*LptB_2_FG-LPS bound. 1,928,889 particles were automatically selected, and then two-dimensional (2D) and three-dimensional (3D) classifications were performed to select consistent particle classes. Finally, 546,301 particles were selected for 3D refinement and a reported 3.1 Å resolution map was generated after post-processing with a *B*-factor of −132 Å^2^. The data processing details are summarised in Supplementary Fig. 5.

The data processing procedures of *sf*LptB_2_FG AMP-PNP bound were similar to the procedures of *sf*LptB_2_FG-LPS bound. 1,762,877 particles were automatically selected, and then 2D and 3D classifications were performed to select consistent particle classes. Finally, 149,178 particles were selected for 3D refinement and a reported 3.2 Å resolution map was generated after post-processing with a *B*-factor of −132 Å^2^. The data processing details are summarised in Supplementary Fig. 9.

### Model building and refinement

Crystal structure of LptB_2_FG of *K. pneumoniae* (PDB code: 5L75) was fitted into the cryo-EM map of *sf*LptB_2_FG LPS bound at 3.7 Å, using UCSF Chimera^54^. The model was then built using COOT^55^. The high-resolution cryo-EM maps allow the side chain assignments according to the bulk side chains. There is a very clear map density in the central cavity of *sf*LptB_2_FG for a rough LPS molecule (Supplementary Figs. 2 and 3). An LPS molecule was built in the density using the Ra-LPS model (PDB:3FXI)^56^. There are densities for detergents near the lateral gates, and two LMNG and four DDM molecules are built in *sf*LptB_2_FG LPS-bound model. Although there are clear densities for the periplasmic domains of LptF and LptG, the periplasmic domains were not able to be built due to the low resolution. The model of *sf*LptB_2_FG-LPS bound was fitted into the 3.1 Å resolution density map of *sf*LptB_2_FGC-LPS. There are densities for LptC TM helix, located between the lateral gate TM1G/TM5F, and LPS located in the cavity. Contrast to the LPS trapped in the *sf*LptB_2_FG, there is no density for the core oligosaccharide of LPS in the *sf*LptB_2_FGC complex, suggesting the core oligosaccharide is flexible (Supplementary Fig. 6). The side chains of *sf*LptB_2_FGC complex are assigned based on the high-resolution cryo-EM density. There is clear density for the periplasmic domains of LptF and LptG and the two domains are fitted well in the density. The density of the periplasmic domain of LptC is not very clear, suggesting that the periplasmic domain is flexible. Detergent molecules (DDM, LMNG) were built near the lateral gates, where LMNG molecule is trapped in the lateral gate TM1G/TM5F (Supplementary Fig. 8). The Crystal structure of LptB_2_FG of *K. pneumonia* (PDB code: 5L75) is fitted in the density of cryo-EM *sf*LptB_2_FGC-AMP-PNP bound map at 3.2 Å. The side chains of *sf*LptB_2_FGC-AMP-PNP bound are assigned based on the high-resolution cryo-EM map, while there is density for the periplasmic domains of LptF and LptG. The TM helix of LptC is no longer at the lateral gate TM1G/TM5F and there is no clear density for LptC periplasmic domain, suggesting that LptC is flexible. The cryo-EM map of *sf*LptB_2_FGC AMP-PNP bound has two clear densities at the active site of LptB (Fig. 4a–d) for two AMP-PNP molecules. There is a density in the cavity through the lateral gate TM1F/TM5G, which we identified it as a DDM molecule (Supplementary Fig. 8). The three structures, *sf*LptB_2_FG LPS bound, *sf*LptB_2_FGC LPS bound and *sf*LptB_2_FGC AMP-PNP bound, were refined using the phenix.real_space_refine in PHINEX^57^. Statistics of 3D reconstruction and model refinement can be found in Supplementary Table 1.

### Reporting summary

Further information on research design is available in the Nature Research Reporting Summary linked to this article.

## Supplementary information

Supplementary Information

Description of Additional Supplementary Files

Supplementary Movie 1

Supplementary Movie 2

Reporting Summary

Source Data

## Data Availability

The atomic coordinates of *sf*LptB_2_FGC-LPS complex, *sf*LptB_2_FG-LPS complex, *sf*LptB_2_FGC-AMP-PNP complex are deposited at Protein Data Bank under access codes 6S8N, 6S8H and 6S8G, respectively. Cryo-EM density maps of *sf*LptB_2_FGC-LPS complex, *sf*LptB_2_FG-LPS complex, *sf*LptB_2_FGC-AMP-PNP complex are deposited at Electron Microscopy Data Bank under access numbers EMD-10125, EMD-10122 and EMD-10121, respectively. The source data underlying Figs. 2d, 6f, 7c and f and Supplementary Figs. 1a–d, 4c, and 4e are provided as a Source Data file. Any other data are available from the corresponding authors upon reasonable request.
