# Supplementary material for: Cryo-EM structures of lipopolysaccharide transporter LptB2FGC in lipopolysaccharide or AMP-PNP-bound states reveal its transport mechanism
Source: Nat Commun. 2019 Sep 13;10:4175. doi: 10.1038/s41467-019-11977-1 (PMC6744409; doi:10.1038/s41467-019-11977-1)
Supplement: Supplementary file 1 — Supplementary Information [file 41467_2019_11977_MOESM1_ESM.pdf]

Supplementary information

Cryo-EM structures of lipopolysaccharide transporter $LptB_2FGC$ in lipopolysaccharide or AMP-PNP-bound states reveal its transport mechanism

X. Tang et al.

Supplementary Figures 1-13 and Supplementary Table 1 and Table 2.

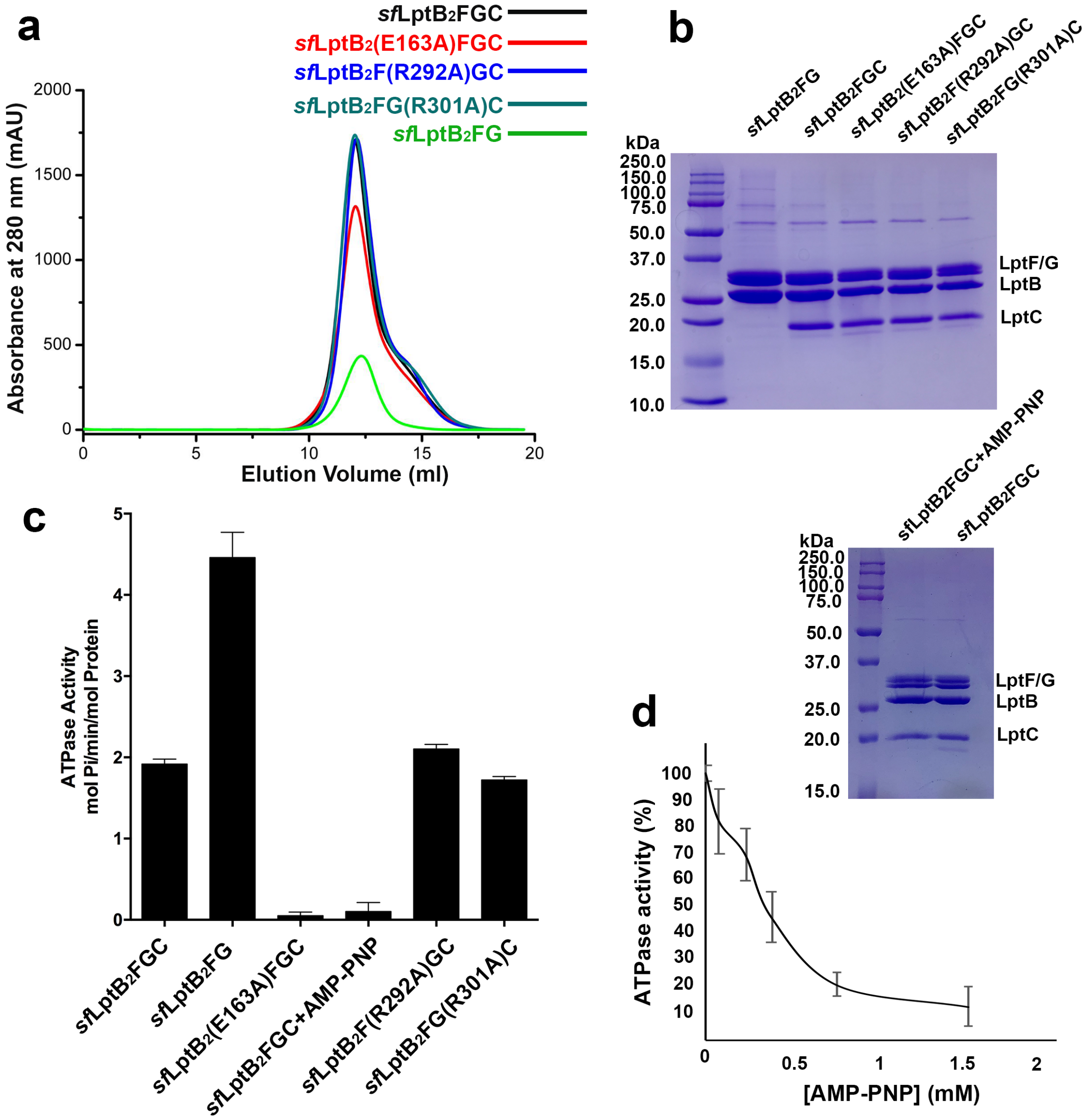

Supplementary Figure 1. ATPase activity of complexes with or without AMP-PNP. a, The size-exclusion chromatography of purified *sf*LptB$_2$FG, *sf*LptB$_2$FGC, *sf*LptB$_2$(E163A)FGC, *sf*LptB$_2$F(R292A)GC and *sf*LptB$_2$FG(R301A)C. **b**, Coomassie brilliant blue staining of purified *sf*LptB$_2$FG, *sf*LptB$_2$FGC, *sf*LptB$_2$(E163A)FGC, *sf*LptB$_2$F(R292A)GC and *sf*LptB$_2$FG(R301A)C. The purified *sf*LptB$_2$FGC was incubated with AMP-PNP for 1 hour at room temperature, then the mixture was purified by a nickel column. After purification of AMP-PNP bound *sf*LptB$_2$FGC, the LptC is detected in complex with LptB$_2$FG. Source data are provided as the source data supplementary fig1b. **c**, The relative ATPase activity of *sf*LptB$_2$FGC, *sf*LptB$_2$FG, *sf*LptB$_2$(E163A)FGC, *sf*LptB$_2$FGC with the AMP-PNP, *sf*LptB$_2$F(R292A)GC and *sf*LptB$_2$FG(R301A)C. Source data are provided as the source data supplementary fig1c. **d**, AMP-PNP inhibits the ATPase activity of *sf*LptB$_2$FGC. Each point represents mean ± s.d. (*n* = 3 biologically independent samples). Source data are provided as the source data supplementary fig1d.

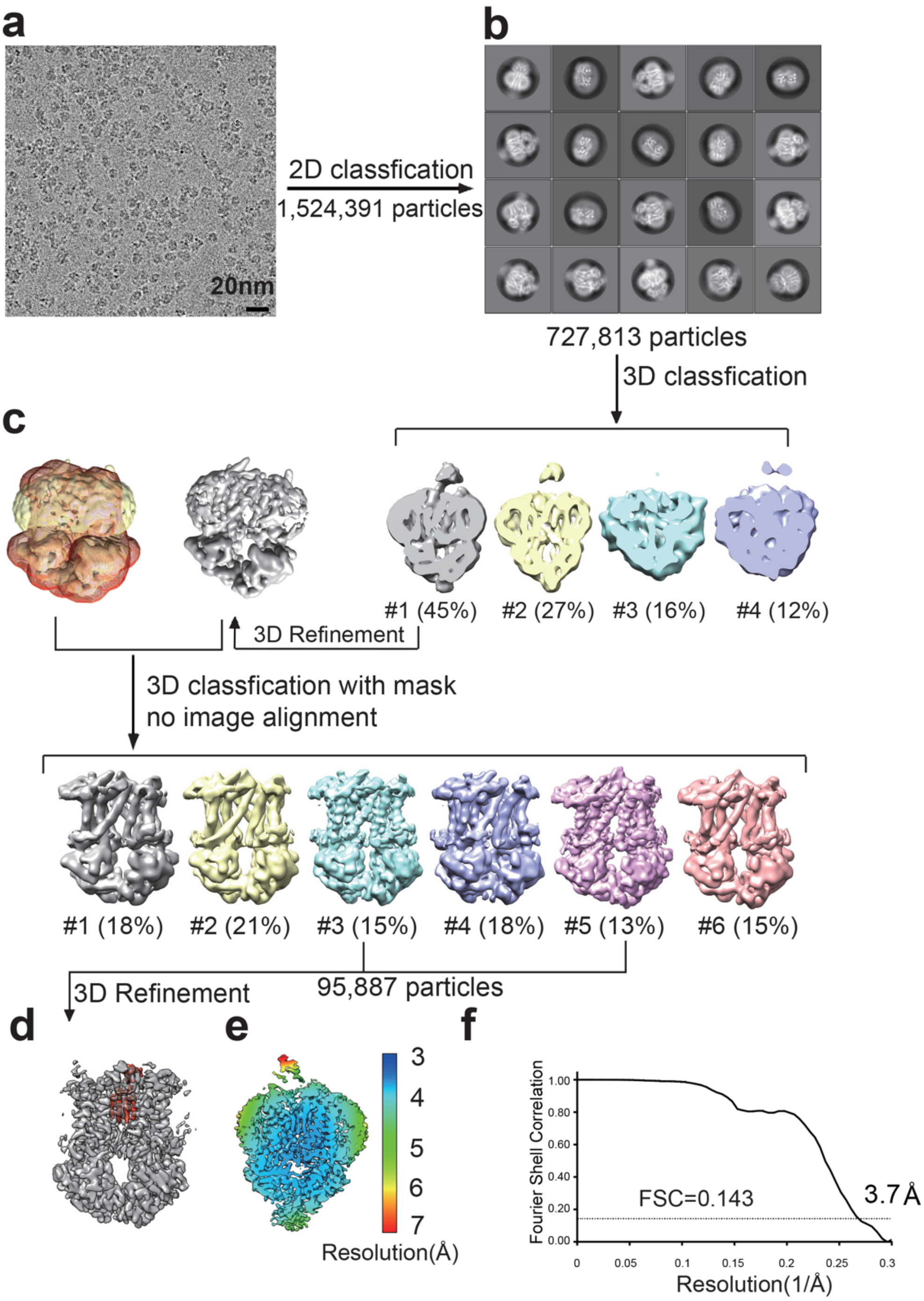

Supplementary Figure 2. Flowchart for cryo-EM single-particle data processing of LPS bound *sf*LptB$_2$FG. a, A micrograph of the single particles after drift correction and dose-weighting. **b**, 2D classifications. **c**, 3D classification and selections. **d**, 3D refinement. cryo-EM density for LPS is coloured in red. **e**, The overall EM maps of the *sf*LptB$_2$FG bound LPS complex are colour coded to indicate the range of resolutions. **f**, Gold-standard FSC curves of the final EM maps.

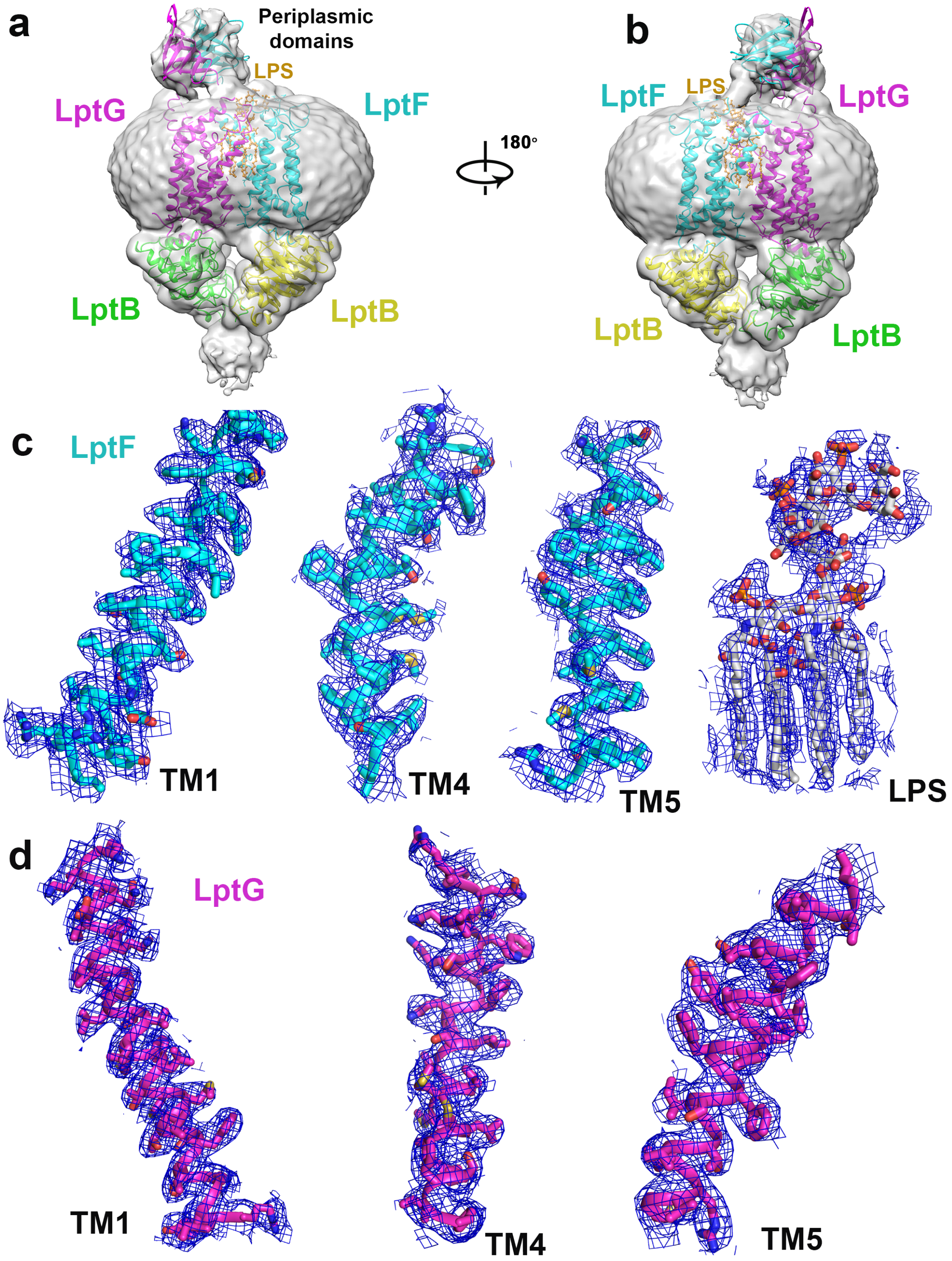

Supplementary Figure 3. Atomic model of *sf*LptB$_2$FG bound LPS fits to its cryo-EM map densities. a, Side view of cryo-EM map of LPS (coloured in orange) bound *sf*LptB$_2$FG with atomic model, showing lipid bilayer and periplasmic domains**. b,** Rotation of 180° along the y-axis relative to the left panel. **c**, Residue side chains of TM1, TM4 and TM5 of LptF are shown in the cryo-EM map densities. LPS is fitted in the map density. **d**, Residue side chains of TM1, TM4 and TM5 of LptG are fitted in the map densities.

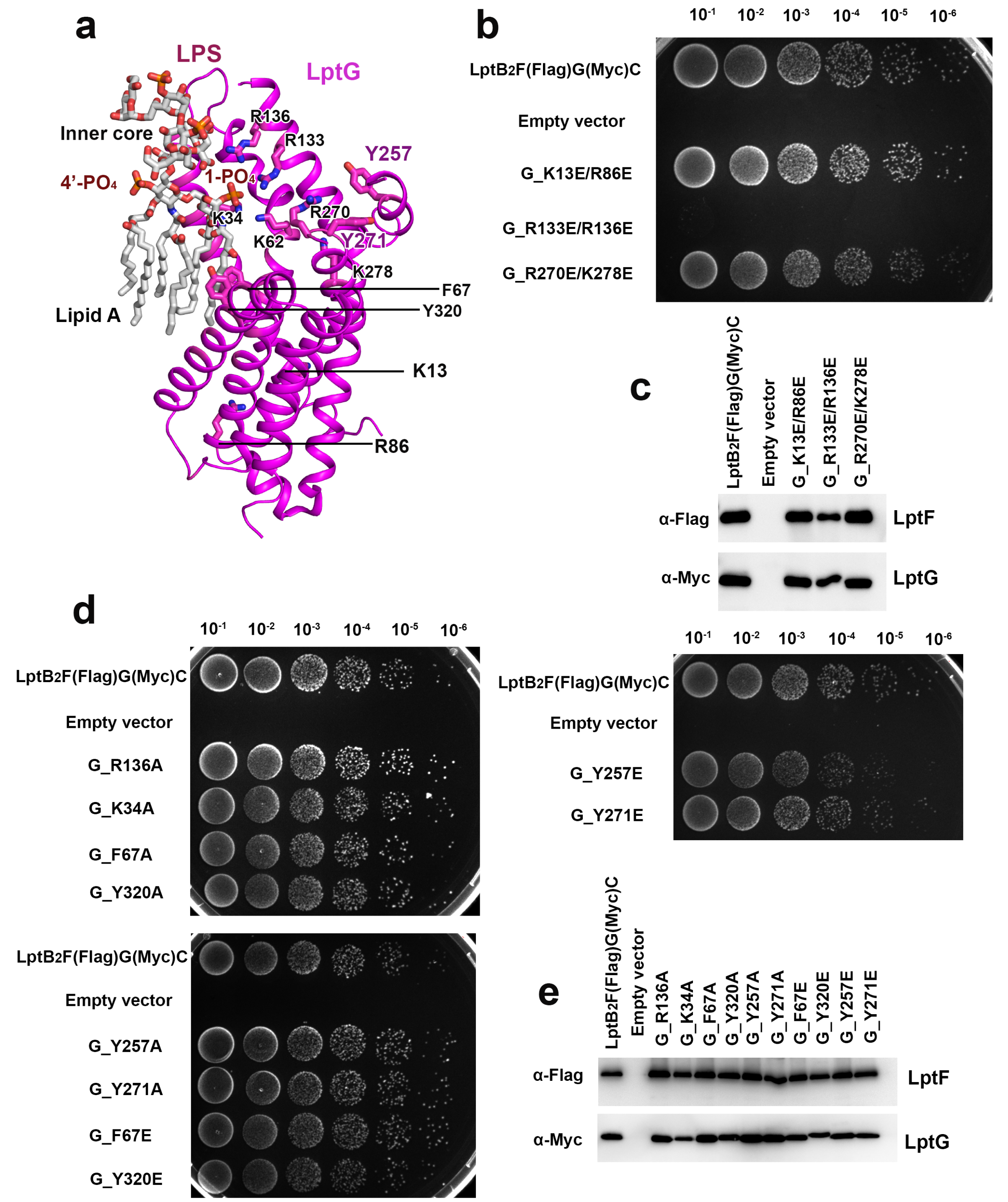

Supplementary Figure 4. Functional assays of LptG residues. a, Residues of LptG and LPS. **b**, Functional assays of LptG mutants. The double mutants K13E/R86E and R270E/K278E has no impact on cell growth. The mutant R133E/R136E caused the cell death. **c**, Protein expression level of LptG mutants was detected by Western blotting. Source data are provided as the source data supplementary fig4c. **d**, Functional assay of single mutants on LptG residues. The mutants of Y257E and Y271E reduces cell growth. **e**, Protein expression level of LptG mutants was detected by Western blotting. Source data are provided as the source data supplementary fig4e.

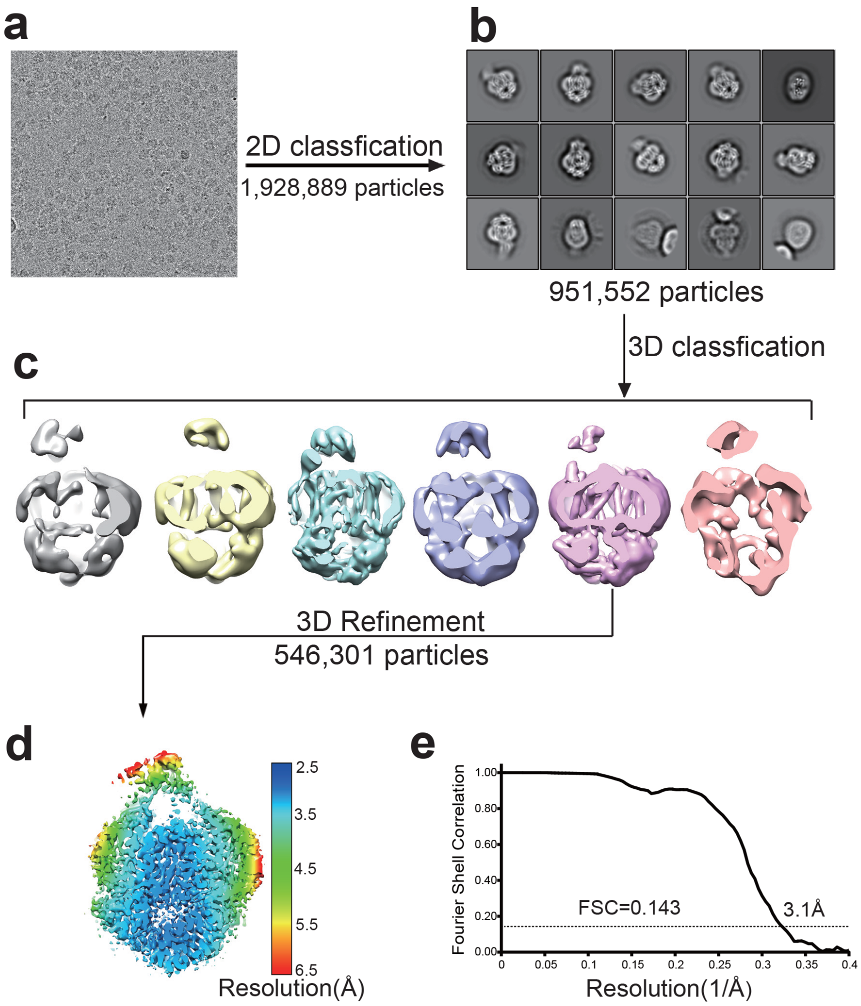

Supplementary Figure 5. Flowchart for cryo-EM single-particle data processing of LPS-bound *sf*LptB$_2$FGC. a, A micrograph of single-particles of *sf*LptB$_2$FGC bound LPS. **b**, 2D classification. **c**, 3D classification, **d**, The overall EM maps of the *sf*LptB$_2$FGC bound LPS complex are colour coded to indicate the range of resolutions. **e**, Gold-standard FSC curves of the final EM maps.

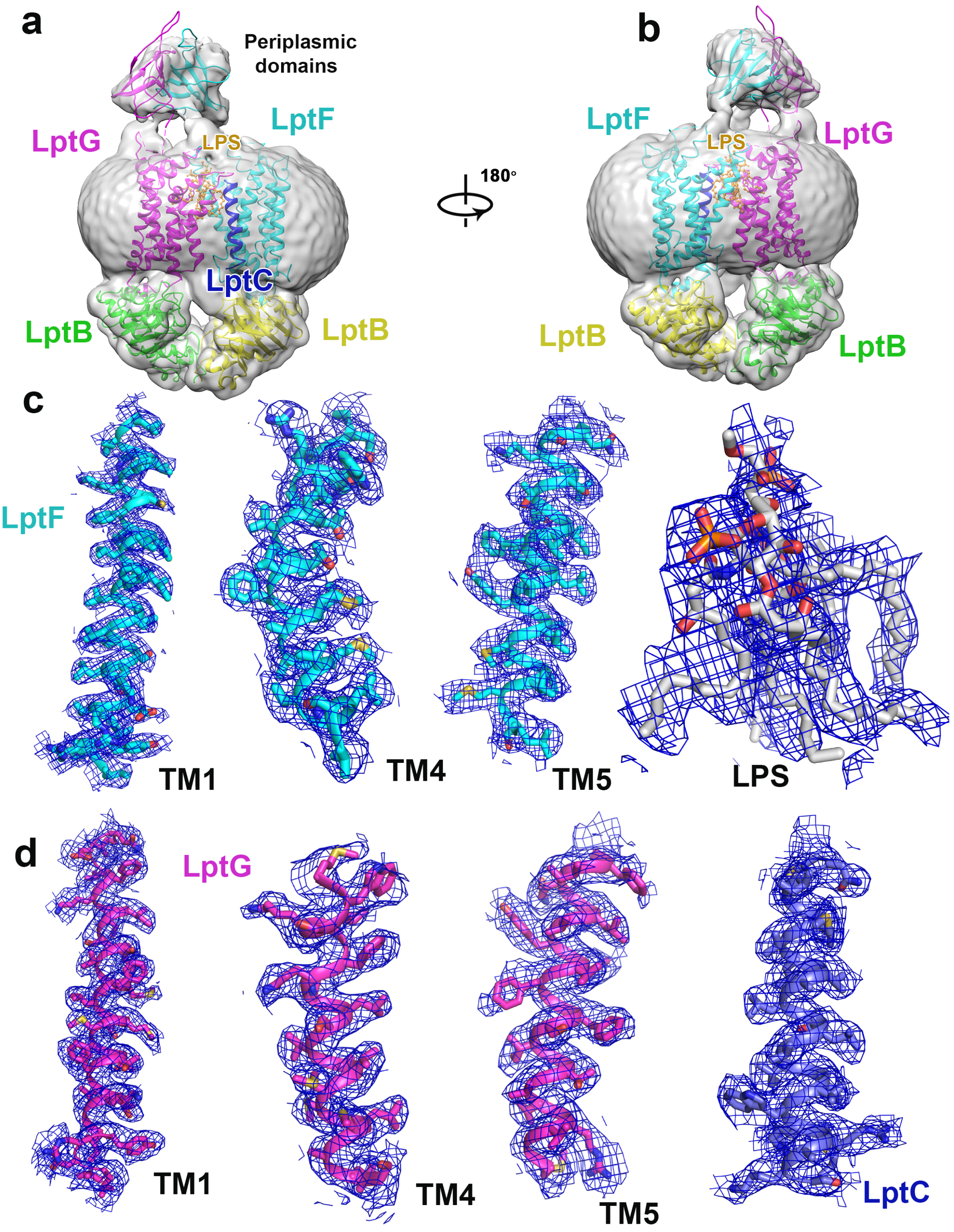

Supplementary Figure 6. Atomic model of *sf*LptB$_2$FGC bound LPS fits to its cryo-EM map densities. a, Side view of cryo-EM map of LPS (coloured in orange) bound *sf*LptB$_2$FGC with atomic model, showing lipid bilayer and periplasmic domains. **b,** Rotation of 180° along the y-axis relative to the left panel. **c**, Residue side chains of TM1, TM4 and TM5 of LptF are shown in the cryo-EM map densities. LPS is fitted in the map density. **d**, Residue side chains of TM1, TM4 and TM5 of LptG and LptC are fitted in the map densities.

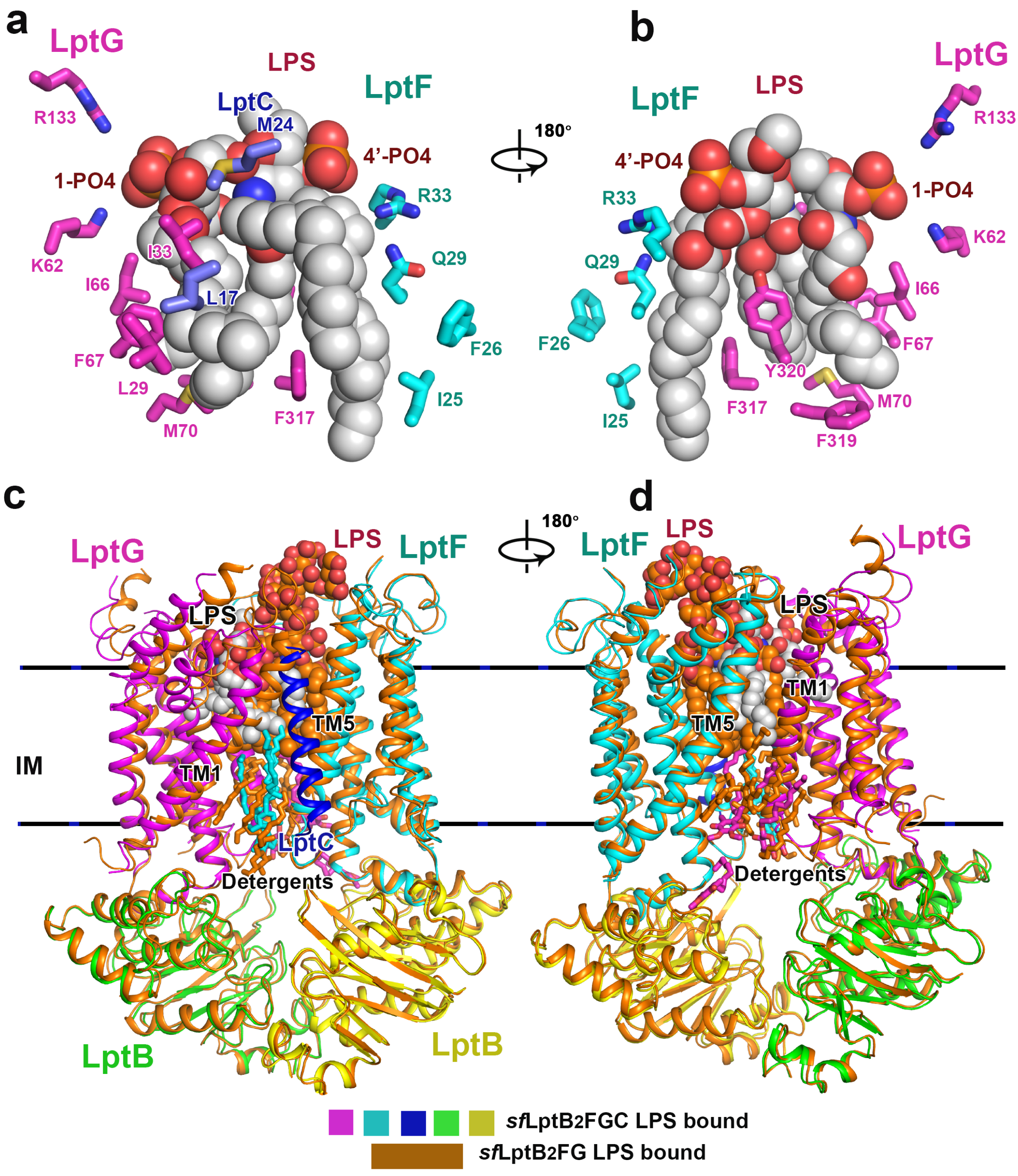

Supplementary Figure 7. LPS is more flexible in *sf*LptB$_2$FGC. The colour scheme for *sf*LptB$_2$FGC is the same as Fig 3. **a**, Residues of *sf*LptB$_2$FGC interact with LPS. **b,** 180 degrees rotation along y-axis of the left panel figure. **c**, Superimposition of *sf*LptB$_2$FGC and *sf*LptB$_2$FG. *sf*LptB$_2$FG is coloured in orange. The carbon atoms of LPS from *sf*LptB$_2$FGC are coloured in grey, while that of the LPS from *sf*LptB$_2$FG are coloured in orange. **d**, 180 degrees rotation along y-axis of the left panel figure.

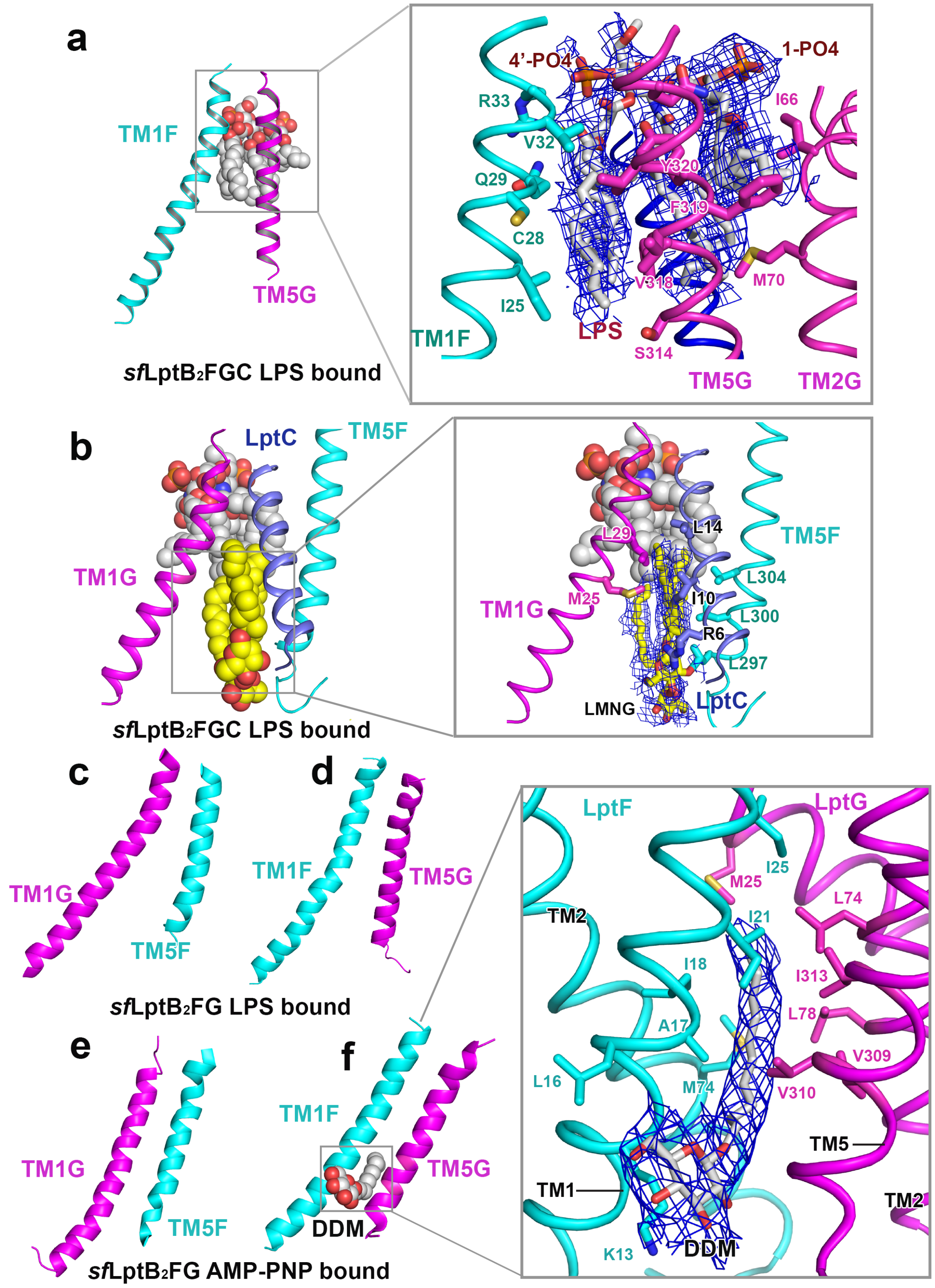

Supplementary Figure 8. Lateral gates of the structures reveal trapped detergents molecules. a, Lateral gates from *sf*LptB$_2$FGC LPS bound. The lateral gates TM1F/TM5G open widely. An acyl tail of LPS is trapped at lateral gate TM1F/TM5G, and a close view of density of LPS. **b**, A LMNG molecule is trapped at the lateral gate TM1G/TM5F from *sf*LptB$_2$FGC LPS bound. a close view of density of LMNG. **c**, **d**: Lateral gates from *sf*LptB$_2$FG bound LPS complex. The gates have

separation at the bottom. **e**, Lateral gates from *sf*LptB_2FGC AMP-PNP bound. The lateral gates are closed. **f**, A DDM molecule is trapped at the lateral gate TM1F/TM5G from *sf*LptB_2FGC AMP-PNP bound. a close view of the density of DDM.

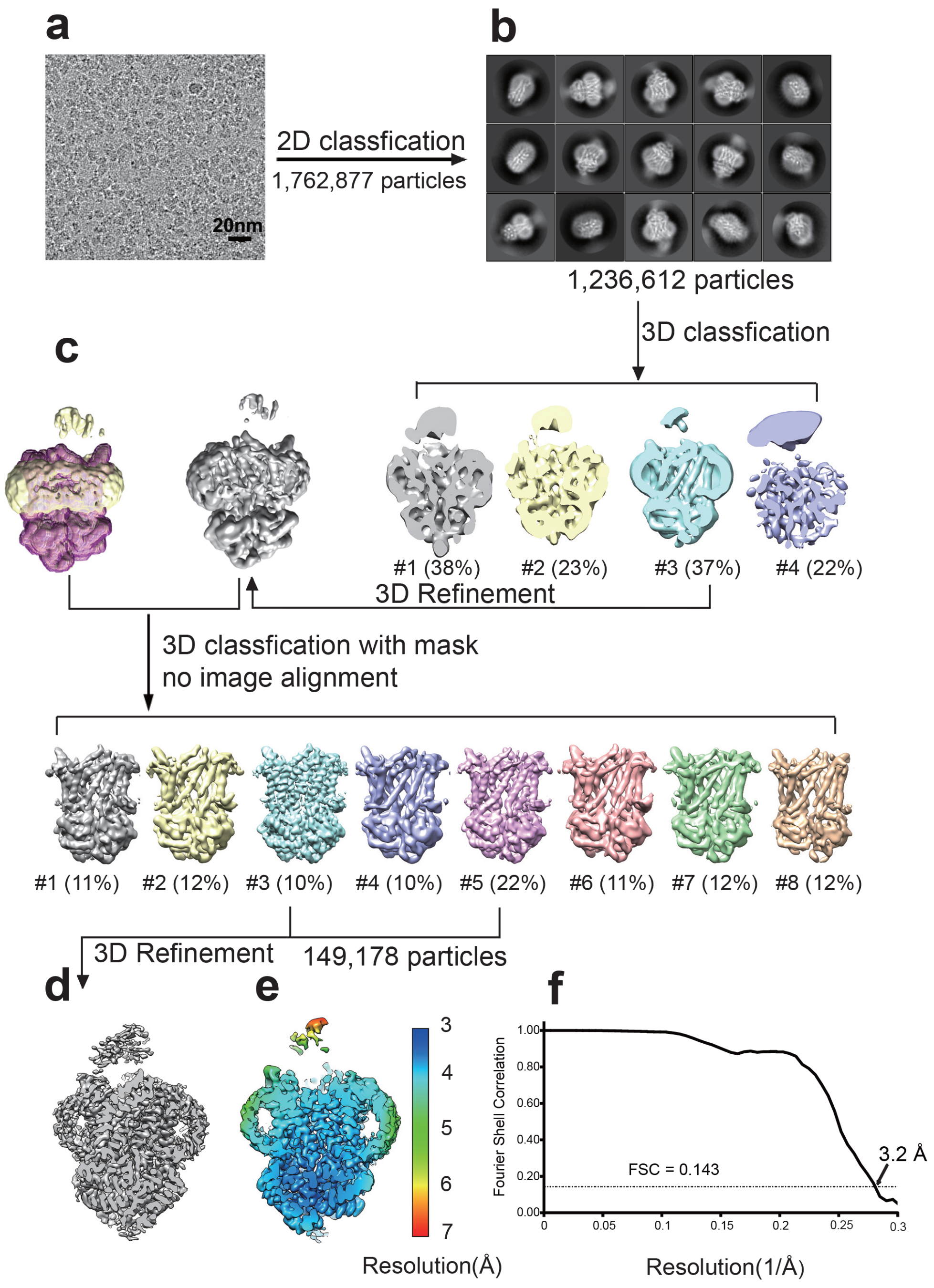

Supplementary Figure 9. Flowchart for cryo-EM single-particle data processing of AMP-PNP bound *sf*LptB$_2$FGC. a, A micrograph of the single particles after drift correction and dose-weighting. **b**, 2D classifications. **c**, 3D classification and selections. **d**, 3D refinement. **e**, The overall EM maps of the *sf*LptB$_2$FGC AMP-PNP bound are colour coded to indicate the range of resolutions. **f**, The gold-standard FSC curve of the final EM maps.

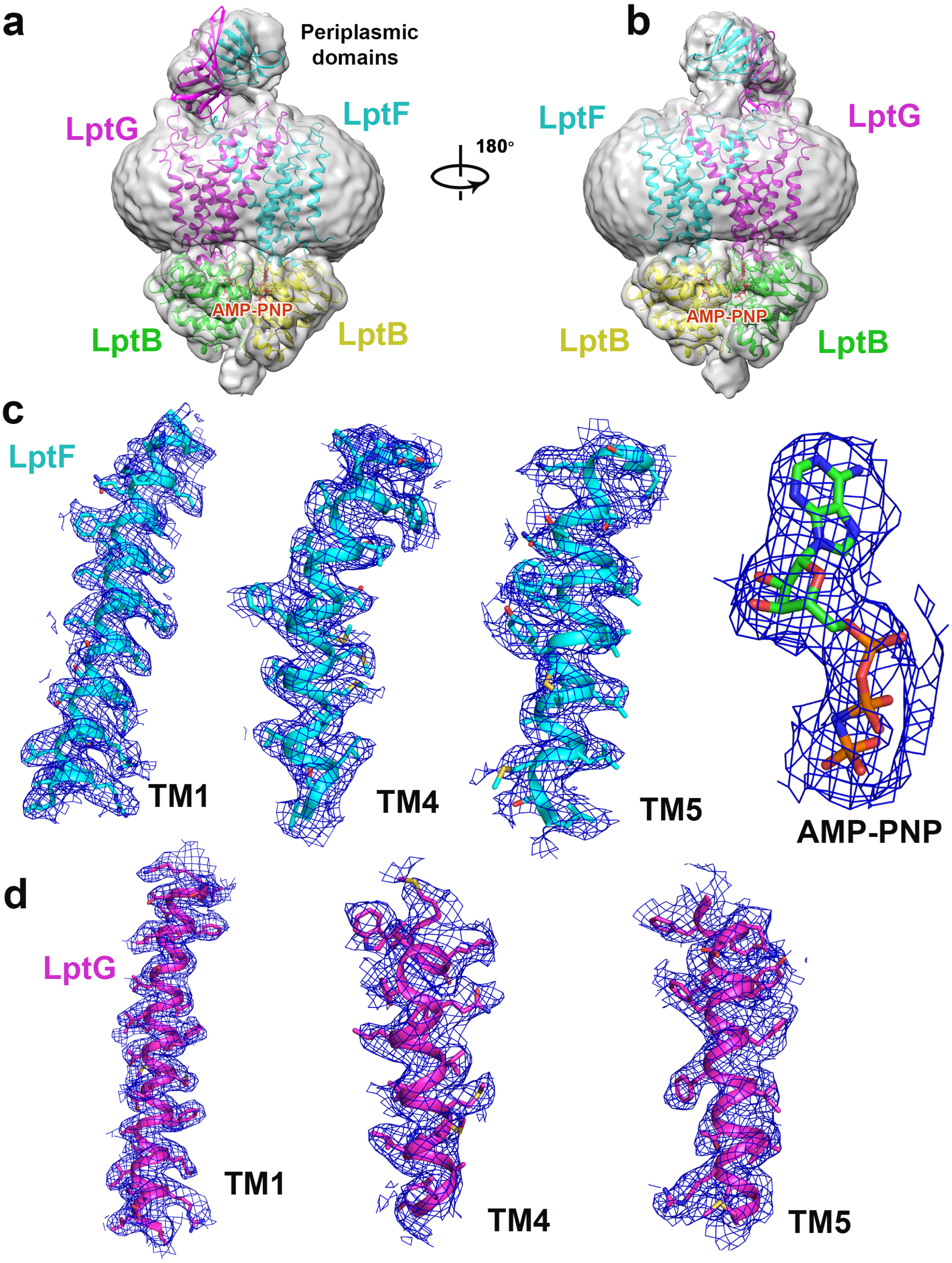

Supplementary Figure 10. Atomic model of *sf*LptB$_2$FGC AMP-PNP bound complex fits to its cryo-EM densities. a, Side view of cryo-EM map of AMP-PNP (coloured in red) bound *sf*LptB$_2$FGC with atomic model, showing lipid bilayer and periplasmic domains**. b,** Rotation of 180° along the y-axis relative to the left panel. **c**, Residue side chains of TM1, TM4 and TM5 of LptF are shown in the cryo-EM densities. AMP-PNP is fitted in the density. **d**, Residue side chains of TM1, TM4 and TM5 of LptG are fitted in the densities.

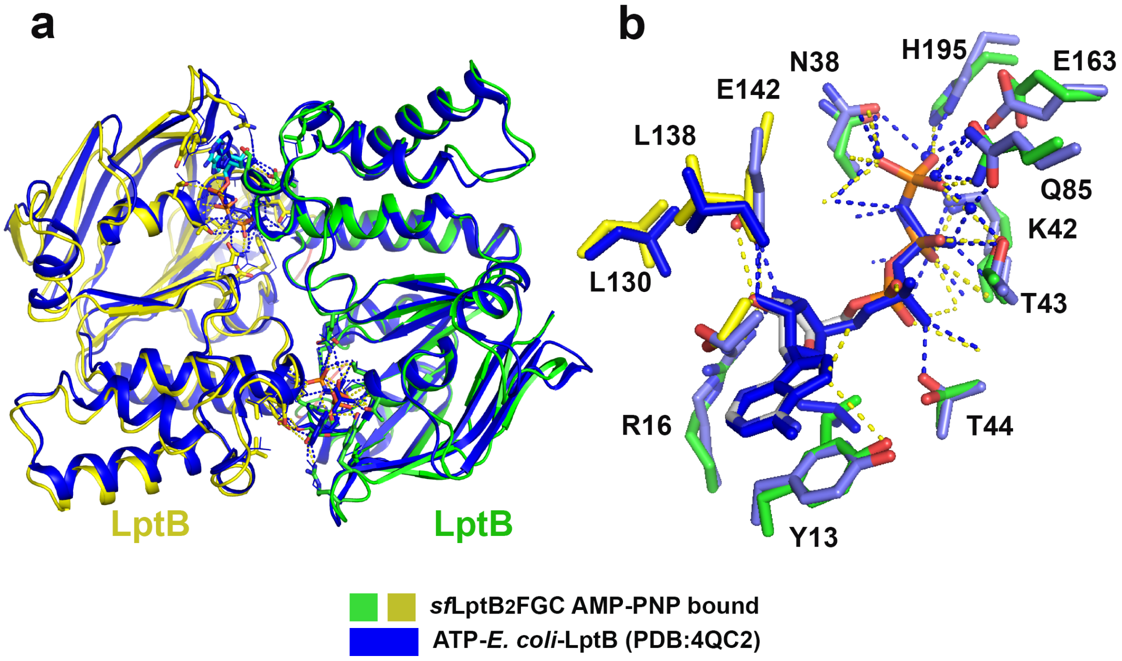

Supplementary Figure 11. AMP-PNP binds to the active site of LptB. a. Superimposition of AMP-PNP bound NBDs of *sf*LptB$_2$FG with ATP *bound E. coli* LptB (PDB:4QC2). The colour scheme of LptB dimer from *sf*LptB$_2$FGC AMP-PNP bound is the same as Fig 1. ATP bound *E. coli* LptB is coloured blue. AMP-PNP is located at the identical position to that of ATP. The side chains of the binding residues of AMP-PNP are at the similar conformations to that of ATP. **b**, Superimposition of binding residues of ATP with AMP-PNP. The AMP-PNP binding residues are at the identical positions to that of ATP.

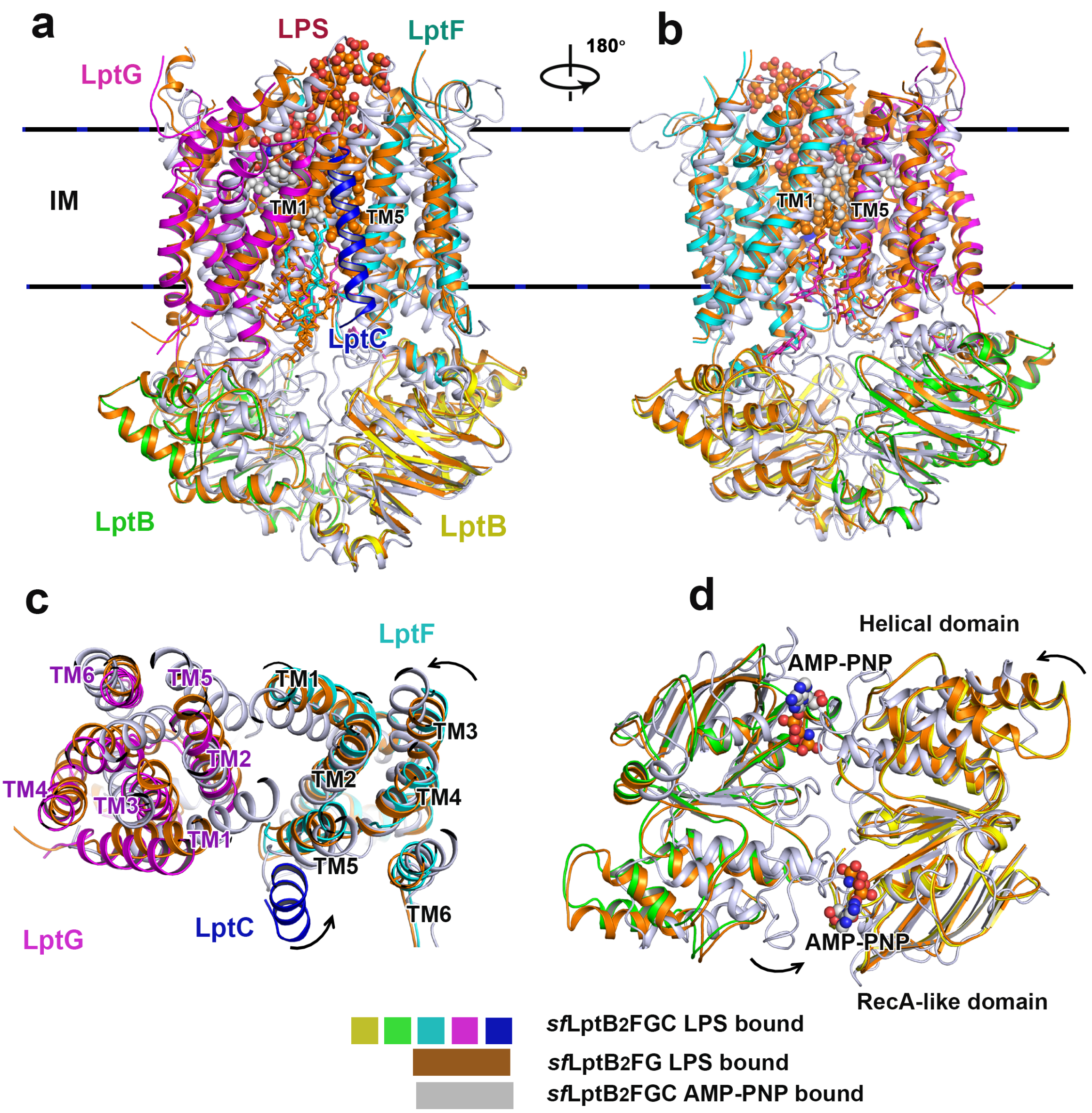

Supplementary Figure 12. Superimposition of the three Cryo-EM structures. a, Superimposition of *sf*LptB_2FGC AMP-PNP bound complex and *sf*LptB_2FG LPS bound complex to *sf*LptB_2FGC LPS bound complex. Significant conformational changes are observed in the NBDs and TMDs. This side view shows the lateral gate TM1G/TM5F**.** *sf*LptB_2FG LPS bound complex and *sf*LptB_2FGC LPS bound complex are in an opened channel conformation, where the channel is open to both periplasm and cytoplasm for LPS binding. *sf*LptB_2FGC bound AMP-PNP is in a closed channel conformation, where the channel is closed. The colour scheme for *sf*LptB_2FGC LPS bound complex is the same as Fig 3, while *sf*LptB_2FG LPS bound complex is in orange, and *sf*LptB_2FGC AMP-PNP bound complex is coloured in grey. **b**, 180 degrees rotation along y-axis of the left panel figure. This side view shows the lateral gate TM1F/TM5G. **c**, Conformational changes of the channel from the open to closed state. The TM helices are rotated at the anti-clock rotation to close the channel from *sf*LptB_2FG(C) LPS

bound complex to *sf*LptB_2FGC AMP-PNP bound complex. **d**, The conformational changes of the dimeric LptB are induced by AMP-PNP.

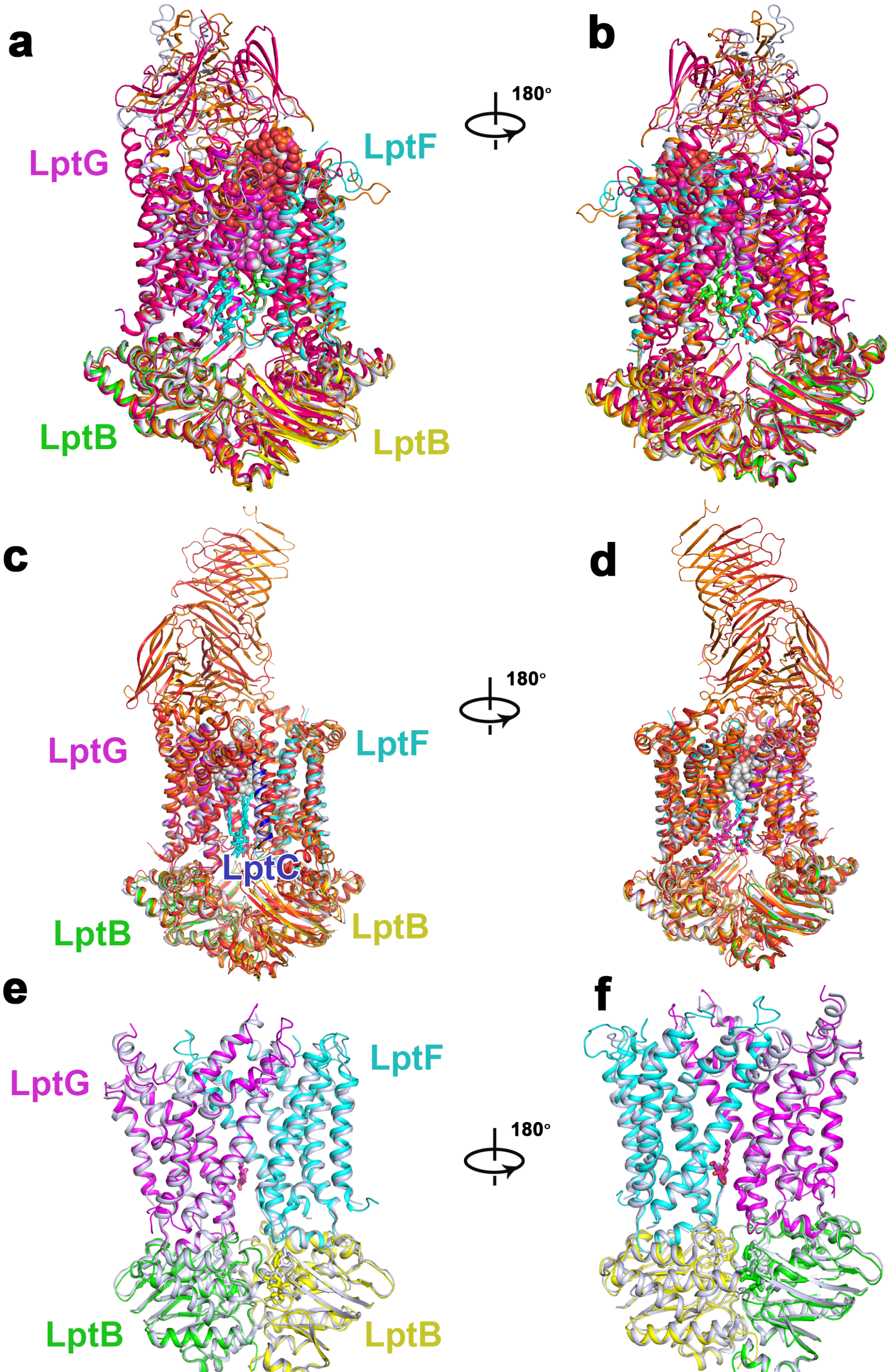

Supplementary Figure 13. Periplasmic domains of LptF and LptG are flexible. a. Superimpositions of LPS-bound *sf*LptB_2FG (coloured same as in fig. 1) to LptB_2FG from *Klebesilla pneumoniae* (PDB code:5L75, orange), LptB_2FG from *Pseudomonas aeruginosa* (PDB code:5X5Y, pink), and LptB_2FG from *E. coli* (PDB code:6MHU, blue white). The cryo-EM structure of *sf*LptB_2FG is similar to the crystal structures of *Klebsiella pneumonia* LptB_2FG, *Pseudomonas aeruginosa* LptB_2FG and cryo-EM structure of *E. coli* LptB_2FG-LPS with RMSD of 1.108 Å over 877 aligned residues, 2.402 Å over 733 aligned residues, and 1.12 Å over 844 aligned residues, respectively.

The periplasmic domains of LptF and LptG are at different conformations, suggesting that the periplasmic domains of LptF and LptG are flexible during LPS transport. **b**, Rotation of 180° along the y-axis relative to the left panel. **c**, Superimpositions of LPS bound *sf*LptB_2FGC (coloured same as in fig. 3) to LptB_2FGC from *Enterobacter cloacae* (PDB code:6MIT, orange), LptB_2FGC from *Vibrio cholerae* (PDB code:6MJP, red) and LptB_2FGC from *E. coli* (PDB code:6MI7, Blue and white). The cryo-EM structure of *sf*LptB_2FGC-LPS bound resembles to the crystal structure of Enterobacter cloacae and Vibrio cholerae and the cryo-EM structure of *E. coli* LptB_2FGC with RMSD of 1.3446 Å over 825 aligned residues, 2.169 Å over 824 aligned residues and 1.1328 Å over 891 aligned residues, respectively. **d**, Rotation of 180° along the y-axis relative to the left panel. **e**, Superimposition of *sf*LptB_2FGC AMP-PNP bound complex and *E. coli* LptB_2FG ADP-vanadate bound complex (PDB code:6MI8, Bluewhite). The overall structures of the two complexes are similar with a RMSD of 1.334 Å over 844 aligned Ca atoms. **f**, Rotation of 180° along the y-axis relative to the left panel.

Supplementary Table 1. Data collection and model statistics.

	sf$LptB_2FG$ LPS	*sf*$LptB_2FGC$ AMP-PNP	*sf*$LptB_2FGC$ LPS
**Data Collection**			
EM equipment		Titan Krios (Thermo Fisher)	
Magnification		49310	
Voltage (kV)		300	
Detector		Gatan K2 Summit	
Pixel size (Å)		1.014	
Electron dose (e-/Å2)		56	
Defocus range (μm)		-1.0 ~ -3.0	
**Reconstruction**			
Software		RELION 3.0	
Number of used particles	95,887	149,178	546,301
Symmetry C1 Final Resolution (Å)	3.7	3.5	3.1
Map sharpening B-factor (Å2)	-104	-132	-136
**Refinement**			
Software	Phenix	Phenix	Phenix
Model composition			
Protein residues	959	957	957
Side chains assigned	959	957	957
AMP-PNP	0	2	0
Detergents	6	2	8
LPS	1	0	1
**R.m.s deviations**			
Bonds length (Å)	0.007	0.009	0.012
Bonds Angle (°)	1.667	1.206	1.761
**Ramachandran plot statistics**			
Preferred (%)	89.84	85.15	91.07
Allowed (%)	9.95	14.74	8.72
Outlier (%)	0.21	0.11	0.21
**PDB code**	6S8H	6S8G	6S8N
**EM map code**	EMD-10122	EMD-10121	EMD-10125

Supplementary table 2. Primer sequences used in the study.

G_R133E_F	CAGGGCGAGCAGATGGCGGAAAACTACCGTGCGCAG
G_R133E_R	TCGCCTGCGCACGGTAGTTTTCCGCCATCTGCTCGC
G_R136E_F	AGCAGATGGCGCGTAACTACGAAGCGCAGGCGAT
G_R136E_R	CCGTACATCGCCTGCGCTTCGTAGTTACGCGCCAT
G_K62E_F	ATACCTTGCTGAGCGTGCCGGAAGATGTGCAGAT
G_K62E_R	GGAAGAAGATCTGCACATCTTCCGGCACGCTCA
G_L26E_F	CACCATCATGATGACACTGTTCATGGAGGTGTCGCTGTCGGGCATTAT
G_L26E_R	ACTTGATAATGCCCGACAGCGACACCTCCATGAACAGTGTCATCATGAT
G_M70E_F	GAAAGATGTGCAGATCTTCTTCCCGGAGGCGGCTCTGCTTGGGGCGTT
G_M70E_R	CAAGCAACGCCCCAAGCAGAGCCGCCTCCGGGAAGAAGATCTGCACAT
G_F67E_F	GAGCGTGCCGAAAGATGTGCAGATCGAGTTCCCGATGGCGGCTCTGC
G_F67E_R	CCCCAAGCAGAGCCGCCATCGGGAACTCGATCTGCACATCTTTCGGC
G_Y320E_F	CGGTATCAGTTTCGGTTTTGTCTTCGAGGTACTGGACCAGATCTTCGG
G_Y320E_R	GCGGGCCGAAGATCTGGTCCAGTACCTCGAAGACAAAACCGAAACTGA
G_Y257E_F	ACTCTCTATCAGCGGTTTGCACAACGAGGTGAAGTATCTGAAGTCGAGC
G_Y257E_R	GACCGCTCGACTTCAGATACTTCACCTCGTTGTGCAAACCGCTGATAGA
G_Y271E_F	GTCGAGCGGTCAGGATGCCGGACGTGAGCAGCTCAACATGTGGAGCAAA
G_Y271E_R	AAGATTTTGCTCCACATGTTGAGCTGCTCACGTCCGGCATCCTGACCG
G_K34E_F	GCTGGTGTCGCTGTCGGGCATTATCGAGTTTGTCGATCAGCTGAAAAA
G_K34E_R	CCGGCTTTTTTCAGCTGATCGACAAACTCGATAATGCCCGACAGCGACA
G_R301A_F	CGTTCATCTTTGGCCCACTGGCGAGCGTACCGA
G_R301A_R	ACGCCCATCGGTACGCTCGCCAGTGGGCCAAA
F_R292A_F	CTGAGCGTGGTTAACCCAGCGCAGGGACGCG
F_R292A_R	CGACAGTACGCGTCCCTGCGCTGGGTTAACCAC
F_D129A_F	GCGTCATCAGGCGGAAGTGTTAGCAGAAGCGAAAGCGAACCCTGG
F_D129A_R	CTAACACTTCCGCCTGATGACGCGATGACCACGGTCCCGCCCAC
F_E265A_F	TGCTCGCGCAGCGCTGAACTGGCGTATCACGTTGGTATTCACCGT
F_E265A_R	GCCAGTTCAGCGCTGCGCGAGCACGATCGGTGTCAGTGTTCCAC
F_R212E_F	GGGAACGGAATTCGAAGGCACTGCATTGTTACGTGATTTCCG
F_R212E_R	TTCGAATTCCGTTCCCTGGTTGAGAGTGACGACCTGGGAG
F_Y230E_F	TTCCAGGATGAACAGGCGATCATTGGTCACCAGGCGGTGGCGCT
F_Y230E_R	ATCGCCTGTTCATCCTGGAAGTCCGTAATGCGGAAATCACGTAAC
F_P139D_F	GAAAGCGAACGATGGCATGGCGGCGCTGGCGCAAG
F_P139D_R	TGCCATCGTTCGCTTTCGCTTCTGCTAACACTTCATCCTGATGACG
F_F149D_F	GCGCAAGGGCAAGATCAGCAAGCGACTAATGGCAGCTCGG
F_F149D_R	TGATCTTGCCCTTGCGCCAGCGCCGCCATGCCAGG
G_W204D_F	GAAGTTTGACCCGGAACATAAAGTCGACCGTCTGTCGCAGGTTGATGA
G_W204D_R	CAGATTCATCAACCTGCGACAGACGGTCGACTTTATGTTCCGGGTCAA
G_I163D_F	GAAAGATGGCAACAACTTCGTCTACGACGAGCGGGTTAAAGGTGACGA
G_I163D_R	ACTCTTCGTCACCTTTAACCCGCTCGTCGTAGACGAAGTTGTTGCCAT

G_L206D_F	TGACCCGGAACATAAAGTCTGGCGTGACTCGCAGGTTGATGAATCTGAT
G_L206D_R	GGTCAGATCAGATTCATCAACCTGCGAGTCACGCCAGACTTTATGTTCCG
G_V209D_F	CATAAAGTCTGGCGTCTGTCGCAGGACGATGAATCTGATCTGACCAAT
G_V209D_R	GGATTGGTCAGATCAGATTCATCGTCCTGCGACAGACGCCAGACTT
G_K13E_F	TGGCGTACTTGACCGCTATATCGGTGAGACTATTTTCACCACCATCATGA
G_K13E_R	TCATCATGATGGTGGTGAAAATAGTCTCACCGATATAGCGGTCAAGTACG
G_R86E_F	GCTTGGTCTTGGGATGCTGGCGCAGGAGAGCGAACTGGTGGTGATGCA
G_R86E_R	AAGCCTGCATCACCACCAGTTCGCTCTCCTGCGCCAGCATCCCAAGAC
G_R270E/K278E_F	CGAGCGGTCAGGATGCCGGAGAGTATCAGCTCAACATGTGGAGCGAGATCTTCCAGCCG
G_R270E/K278E_R	ACAGATAGCGGCTGGAAGATCTCGCTCCACATGTTGAGCTGATACTCTCCGGCATCCTG
G_R136A-F	AGCAGATGGCGCGTAACTACGCGGCGCAGGCGAT
G_R136A-R	CCGTACATCGCCTGCGCCGCGTAGTTACGCGCCAT
G_K34A_F	ATCGCGTTTGTCGATCAGCTGAAAAAAGCCGGG
G_K34A_R	CGACAAACGCGATAATGCCCGACAGCGAC
G_F67A_F	CAGATCGCGTTCCCGATGGCGGCTCTGCTTG
G_F67A_R	GGGAACGCGATCTGCACATCTTTCGGCACGCTC
G_Y320A_F	CTTCGCGGTACTGGACCAGATCTTCGGCC
G_Y320A_R	CAGTACCGCGAAGACAAAACCGAAACTGATACCGG
G_Y257A_F	CAACGCGGTGAAGTATCTGAAGTCGAGCGGTCAGG
G_Y257A_R	CTTCACCGCGTTGTGCAAACCGCTGATAGAGAGTG
G_Y271A_F	GACGTGCGCAGCTCAACATGTGGAGCAAAATCTTCCAGC
G_Y271A_R	GCTGCGCACGTCCGGCATCCTGACCGCTC
Shigella_lptB_EcoRI_F	ATATGAATTCATGGCAACATTAACTGCAAAGAACCTTGC
Shigella_lptB_KpnI_8.His_R	ATATGGTACCTCAGTGATGGTGATGGTGATGGTGATGGAGTCTGAAGTCTTCCCCAAGGTATACAC
Shigella_lptFG_KpnI_F	ATATGGTACCTTTTTACGGGCGTATTTAAAGTGATAATC
Shigella_lptFG_XbaI_R	ATATTCTAGATTACGATTTTCTCATTAACAGCCACAG
Shigella_lptBFG_linerize_F	TCAGCCTTAAGAATTCAGAAGGGTAATTAATTCGTTATGG
Shigella_lptBFG_linerize_R	CTTTGGCTTCCATATGCTGTTTCCTGTGTGAAATTGTTATCCGCTCACA
Shigella_lptC_F	ACAGCATATGGAAGCCAAAGGGCAATCGATATGAGTAAAGCCAG
Shigella_lptC_R	ACAGCATATGGAAGCCAAAGGGCAATCGATATGAGTAAAGCCAG
