# Supplementary material for: Cryo-EM structures of lipopolysaccharide transporter LptB2FGC in lipopolysaccharide or AMP-PNP-bound states reveal its transport mechanism
Source: Nat Commun. 2019 Sep 13;10:4175. doi: 10.1038/s41467-019-11977-1 (PMC6744409; doi:10.1038/s41467-019-11977-1)
Supplement: Supplementary file 2 — Description of Additional Supplementary Files [file 41467_2019_11977_MOESM2_ESM.pdf]

Description of Additional Supplementary Files

File name: Supplementary Movie 1

Description: A rotational motion from the *sf*LptB_2FGC LPS bound structure to the *sf*LptB_2FGC AMP-PNP bound structure from a side view. A rotational motion from the *sf*LptB_2FGC LPS bound structure to the *sf*LptB_2FGC AMP-PNP bound structure and then return to the LPS bound state from the side view of lateral gate TM1G/TM5F. The sfLptB2FGC LPS bound structure is in the same colour scheme of Fig3, except TM helix of LptC in magenta as LptG. The sfLptB2FGC AMP-PNP bound structure in orange. The LptB2FGC is rotated at counter-clockwise direction to close the channel and push LPS out of the cavity, while the LptB2FGC is rotated at clockwise direction to open the channel to extract LPS from the IM.

File name: Supplementary Movie 2

Description: A rotational motion from the *sf*LptB_2FGC LPS bound structure to the *sf*LptB_2FGC AMP-PNP structure from a side view of 180° rotation along y-axis of movie 1. A rotational motion from the *sf*LptB_2FGC LPS bound structure to the *sf*LptB_2FGC AMP-PNP structure and then return to the LPS bound state from the side view of lateral gate TM1F/TM5G. The sfLptB2FGC LPS bound structure is in the same colour scheme as that of Fig3. The sfLptB2FGC AMP-PNP bound structure in orange. The LptB2FGC is rotated at counter-clockwise direction to close the channel and push LPS out of the cavity, while the LptB2FGC is rotated at clockwise direction to open the channel to extract LPS from the IM.
